# CHIKV mRNA vaccines encoding conserved structural/envelope proteins confer broad cross-lineage protection against infection

**DOI:** 10.1038/s41392-025-02182-2

**Published:** 2025-03-28

**Authors:** Xiaoming Liang, Yanan Zhou, Yun Yang, Qianqian Li, Junbin Wang, Bai Li, Hao Yang, Cong Tang, Wenhai Yu, Haixuan Wang, Qing Huang, Hongyu Chen, Yuhuan Yan, Ran An, Dongdong Lin, Wenqi Quan, Yong Zhang, Yanwen Li, Xuena Du, Yuxia Yuan, Longhai Yuan, Jian Zhou, Qiangming Sun, Youchun Wang, Shuaiyao Lu

**Affiliations:** 1 Yunnan Key Laboratory of Cross-Border Infectious Disease Control and Prevention and Novel Drug Development, Institute of Medical Biology, Chinese Academy of Medical Sciences and Peking Union Medical College, Kunming, China; 2 State Key Laboratory of Respiratory Health and Multimorbidity, Beijing, China; 3 Key Laboratory of Pathogen Infection Prevention and Control (Peking Union Medical College), Ministry of Education, Beijing, China; 4 Yunnan Provincial Key Laboratory of Vector-borne Diseases Control and Research, Kunming, China

**Keywords:** Vaccines, Vaccines

## Abstract

With the broad spread of the chikungunya virus (CHIKV), there is an increasing demand for more effective and broadly protective vaccines. Here, we designed CHIKV mRNA vaccines containing full-length structural proteins or part of structural proteins (envelope proteins) based on conserved sequences from 769 viral strains encompassing four lineages. The vaccine induced strong cellular and humoral immune responses in BALB/c mice and provided robust protection. Immunization of BALB/c mice with either of the two vaccines induced high levels of neutralizing antibodies against pseudoviruses from four distinct lineages, highlighting their potential for broad cross-lineage protective efficacy. Immunoglobulin repertoire analysis revealed two important BCR V-J gene combinations, IgHV1-4-IgHJ3 and IgHV1-4-IgHJ2, and lineage-specific immunity analysis revealed significant upregulation of TCRs containing V19 and V20. BCR and TCR immunodiversity may be a potential reason for the broad-spectrum protection against CHIKV afforded by the vaccine. In A129 mice, it elicited lower levels of neutralizing antibodies but prevented mouse mortality and cleared chronic infection. In the rhesus macaque model, both vaccines elicited a certain level of humoral and cellular immune responses and protected the rhesus macaques from the CHIKV challenge. In conclusion, the results from both mouse and rhesus macaque models indicate that the vaccine could be a candidate for clinical use against CHIKV.

## Introduction

Chikungunya virus (CHIKV) is a globally distributed arthritogenic alphavirus transmitted by Aedes mosquitoes, causing sporadic outbreaks occurring every 2–50 years, particularly in tropical regions. Since its first discovery in 1952,^1^ CHIKV has sporadically emerged in Africa and spread to tropical and temperate regions across other continents. CHIKV cases have been reported in more than 100 countries, with cumulative reports of more than 10 million cases, posing a risk to an estimated 1.3 billion people in transmission-prone areas.^2,3^ In 2023, CHIKV reemerged in the Americas, causing ~350,000 infections and spreading to new countries.^4^ The pandemic potential of CHIKV has long been recognized, leading to its prioritization by the Coalition for Epidemic Preparedness Innovations (CEPI) for vaccine development. In 2018, CHIKV was included in the World Health Organization’s list of priority pathogens for vaccine research and development.^5^ With the globalization of travel and trade, changes in the global climate, and the expansion of the habitat range of *Aedes albopictus* mosquitoes, the trend of a CHIKV pandemic is becoming increasingly evident.^6,7^

CHIKV has broad tissue tropism, and the key cell in which CHIKV replicates in vivo is probably the fibroblast. There are a few more cell types in which CHIKV does not replicate well, such as hepatocytes,^8^ renal cells,^9^ also does not normally infect lungs or the testes, etc. Brain infection is also rare—except in neonates. Upon infection, individuals develop severe viremia, followed by systemic infection of various tissues, leading to typical symptoms, including fever, headache, stiffness, photophobia, rash, and severe joint pain.^10^ The disease can persist for several months,^11–13^ significantly impacting quality of life.^14–17^ Children may occasionally experience neurological symptoms such as seizures, impaired consciousness, blindness due to optic neuritis, and acute flaccid paralysis.^18^ Chikungunya fever (CHIKVF) was previously considered a self-limiting disease, but with the spread of CHIKV, an increasing number of severe atypical symptoms have been reported.^19^

CHIKV has diverged into four lineages, including the West African lineage, the East/Central/South African (ECSA) lineage, and the Asian lineage, and the appearance of the Indian Ocean lineage (IOL) has led to the gradual spread of CHIKV, which was originally confined to tropical regions and transmission by *Aedes aegypti* mosquitoes, to temperate regions inhabited by *Aedes albopictus* mosquitoes.^5,20,21^

Like other alphaviruses, CHIKV is a single-stranded positive-sense RNA virus with a genome of 11.8 kb that includes two ORFs encoding the structural polyprotein C-E3-E2-6k-E1 and nonstructural proteins nsp1, nsp2, nsp3, and nsp4. The E2 protein forms a complex with the E1 protein, and three E2-E1 complexes form a trimeric envelope protein.^22,23^ E2 and E1 are the main antigenic epitopes, with certain residues of E2 and E1 involved in receptor binding and E1 associated with membrane fusion, while E3 acts as a chaperone protein.^23^ Research has shown that there are differences in the neutralizing and binding properties of antiserum from recovered individuals against the virus among different lineages.^24^ However, overall, antiserum has good protective efficacy. Within the Alphavirus genus, the fusion region of the E1 protein is highly conserved,^25^ and the main neutralizing epitopes of the E2 protein show high homology,^25,26^ resulting in cross-protective ability of neutralizing antibodies. Currently, there are no specific drugs for treating CHIKV, and symptomatic treatment, such as nonsteroidal anti-inflammatory drugs used to alleviate pain,^27^ which do not have any preventive effect, is the main approach. There are currently few available vaccines, with only one live attenuated vaccine approved by the FDA. However, live-attenuated vaccines are considered to have safety concerns, causing adverse events, and the potential for virus replication leading to transmission through *Aedes mosquitoes*. VLA1553 has recently been approved by the FDA and has shown encouraging immunogenicity and protective efficacy,^28^ but it is associated with paw swelling in mice and systemic virus replication in immunocompetent mice, nonhuman primates, and humans. Moreover, in a recent phase III clinical trial, 57.1% of patients experienced systemic adverse events, including muscle and joint pain, across three different vaccine batches.^4,29^ There is an urgent need for a vaccine with high efficacy, safety, and production capacity to address the threat of CHIKV. mRNA vaccines have demonstrated significant advantages during the severe acute respiratory syndrome coronavirus 2 (SARS-CoV-2) pandemic and have emerged as promising next-generation vaccine platform.^30,31^ They possess the full functionality of posttranslational modifications and utilize host cells as factories, allowing accurate expression, folding, and secretion of antigenic proteins to mimic the virus.^32^ Notably, mRNA vaccines have short development cycles, induce rapid immune responses, and thus exhibit considerable potential for responding to emergent situations.^33^

The CHIKV vaccine candidates currently under investigation for clinical trials include attenuated live vaccines, virus-like particle (VLP) vaccines, viral vector vaccines, and mRNA vaccines.^34^ mRNA-1388 is the first mRNA vaccine targeting CHIKV. Phase I clinical trials demonstrated that it elicited high levels of persistent neutralizing antibodies in healthy individuals from non-endemic regions, and it also highlighted the safety of mRNA vaccines; however, its design is based on a single strain. A preclinical study utilizing an mRNA vaccine employed the E2-E1 soluble region as the antigen and showed that the mRNA vaccine could induce a more robust cellular immune response compared to subunit vaccines. Another study indicated that modified mRNA vaccines exhibited superior immunogenicity compared to their unmodified counterparts. Although the main antigenic epitopes of CHIKV are considered conserved,^26^ CHIKV may exhibit a high propensity for mutation, and there is considerable variation in the reactivity of sera from recovered individuals infected with viruses from different lineages.^24^

## Results

### The vaccines mCV-1 and mCV-2 achieve high levels of expression in vitro

**Fig. 1:**
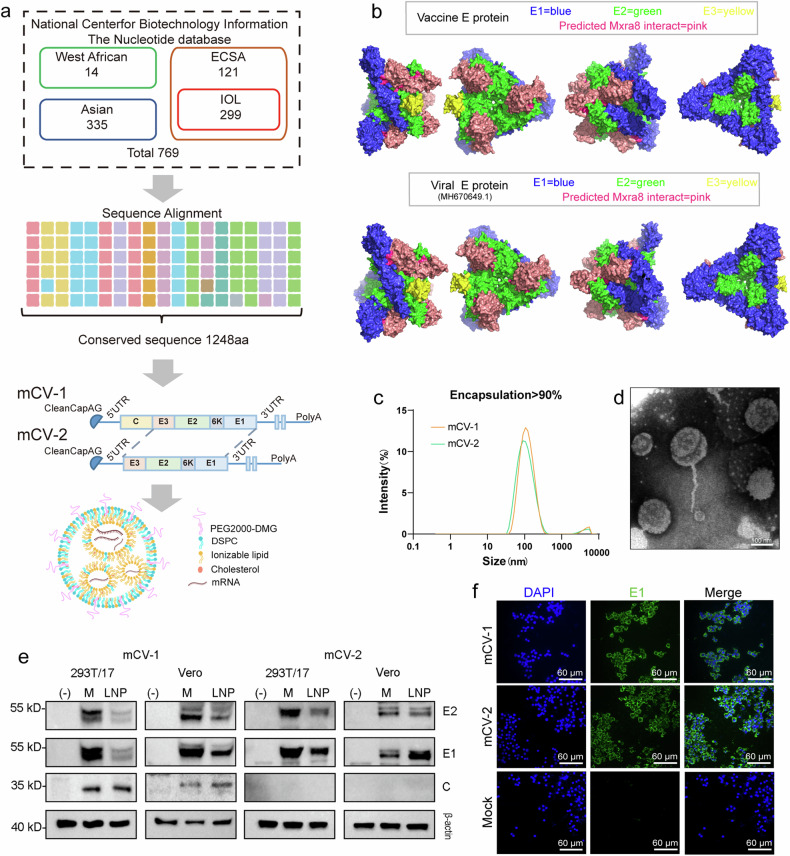
Vaccine design, preparation, and in vitro expression. **a** Vaccine design. A total of 769 strains were identified from the NCBI database, including 14 from the West African lineage, 335 from the Asian lineage, 121 from the East, Central, and South African lineage, and 299 from the Indian Ocean lineage. Conserved sequences were selected for further analysis; **b** Utilize Alpha-Fold3 for structural prediction and receptor interaction prediction of the E protein in vaccine sequences and viral sequences (MH670649.1); **c** Dynamic light scattering (DLS) particle size distribution; **d** Transmission electron microscopy (TEM) images; **e** E1, E2, C protein, and β-actin Western blot images; (−) indicates the negative control, and M indicates the control transfected with a commercial kit; **f** Immunofluorescence experiments were conducted to detect the distribution of the E1 protein, using nontransfected cells as the mock condition

To enhance the cross-protective efficacy of the vaccine, we employed the strategy of identifying conserved sequences, similar to previous studies.^35^ We retrieved full-length structural protein sequences from 769 strains belonging to the four different lineages from the NCBI database and constructed a phylogenetic tree (Supplementary Fig. 1a) consisting of four branches representing each lineage. The 769 full-length structural protein sequences were aligned, and 1248 conserved amino acid positions were identified (The selection of non-conserved regions included the most frequently occurring amino acids among the various strains). These conserved amino acids were then sequentially assembled into a novel full-length structural protein sequence, designated candidate vaccine protein sequence 1 (Fig. 1a). Additionally, a portion of the envelope protein E3-E2-6K-E1 was selected as candidate vaccine protein sequence 2. To enhance mRNA expression, we optimized the candidate protein sequences according to human codon usage bias, resulting in two vaccine sequences, mCV-1 and mCV-2. Based on the structural prediction and receptor interaction analysis using AlphaFold3,^36^ it is shown that the assembly of the E protein sequence based on individual amino acids does not exhibit significant changes in structure or receptor binding compared to the viral E protein (Fig. 1b, Supplementary Fig. 1b).

Following previously described methods, mRNA-lipid nanoparticles (LNPs) were prepared using a LNP delivery system, achieving encapsulation rates exceeding 90%, as further confirmed by agarose gel electrophoresis (Supplementary Fig. 1c). Dynamic light scattering (DLS) revealed an average particle size of approximately 100 nm (Fig. 1c), while transmission electron microscopy demonstrated a uniform particle size distribution (Fig. 1d). To validate the in vitro expression achieved by mRNA-LNPs, purified mRNA from both variants was transfected into 293T/17 cells and Vero cells using LNPs, while naked RNA transfection was conducted as a control using a commercial kit. Western blot experiments were used to detect various structural proteins (C, E2, and E1) in both 293T/17 and Vero cells (Fig. 1e). Furthermore, to verify the intracellular expression of antigens, immunofluorescence assays targeting the E1 protein were performed in Vero cells (Fig. 1f), resulting in successful detection of the E1 protein both intracellularly and on the cell membrane, as clearly visualized by confocal microscopy (Supplementary Fig. 1d).

### The mCV-1 and mCV-2 vaccines strongly trigger humoral immune responses and the production of cross-protective neutralizing antibodies

**Fig. 2:**
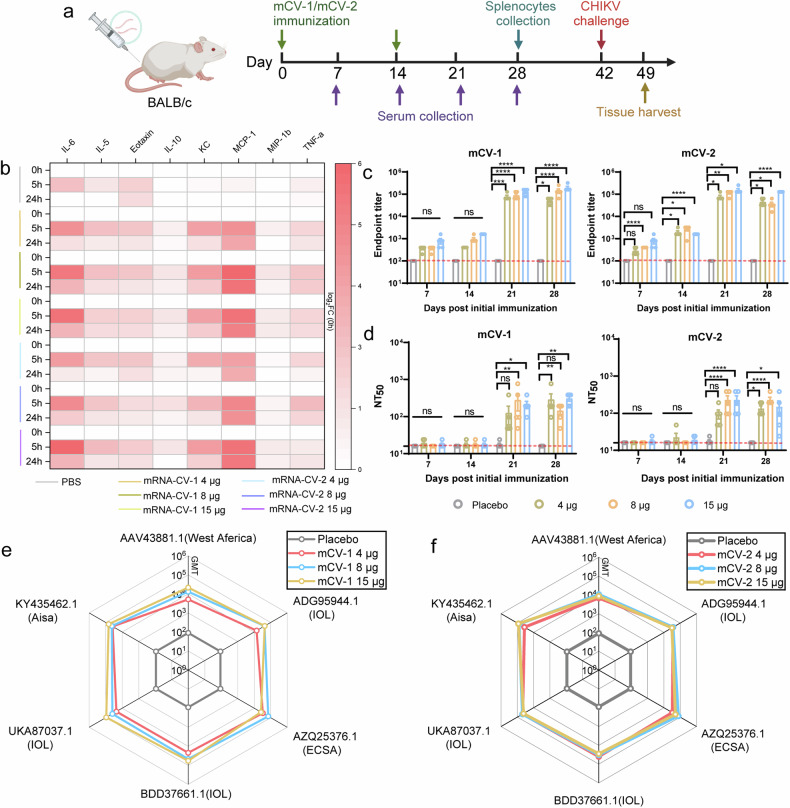
mCV-1 and mCV-2 induced strong immune responses in BALB/c mice. **a** The vaccination and sample collection timeline, the collection of serum for cytokine quantification at 5 and 24 h after the initial immunization, serum collection on days 7, 14, 21, and 28, spleen collection on day 28, and challenge experiments on day 42; **b** The changes in relevant cytokine levels at 5 and 24 h post-immunization, normalized to the levels at 0 hours and logarithmically transformed; log_2_FC (0 h) means the ratio of cytokine level at 5 or 24 h to that at 0 h; **c** and **d** The titers of binding antibodies (**c**) and neutralizing antibodies against adapted strains (**d**) at different time points; e-f, GMTs of pseudovirus neutralizing antibodies against various strains at 28-day post-immunization for mCV-1 (**e**) and mCV-2 (**f**). The data are presented as the mean ± SEM (*n* = 5), and each symbol represents a mouse. Statistical analysis was conducted using one-way ANOVA and Tukey’s multiple comparison tests for bar graphs; ***p < 0.001; ****p < 0.0001; ns, not significant

To evaluate the immunogenicity of the vaccines, immunization experiments were conducted in BALB/c mice in which three doses of mCV-1 or mCV-2—low dose (4 μg), medium dose (8 μg), or high dose (15 μg)—were administered via intramuscular injection, with empty LNPs serving as the control. During the observation period after immunization, no abnormalities in body temperature or weight were observed (Supplementary Fig. 1e, f), nor were there any local adverse reactions. Muscle and liver tissues were collected 24 h post-immunization, and the expression of antigen proteins was detected (Supplementary Fig. 2a). A booster immunization at the same dose was administered 14 days after the initial immunization (Fig. 2a). To assess the immune responses of the mice, serum samples were collected at 5 and 24 hours post-primary immunization for the quantification of immune response-related cytokines (Fig. 2b). Most cytokine levels significantly increased at 5 h and decreased markedly by 24 h, with IL-6, MCP-1, and TNF-α remaining at high levels at 24 h. IL-5 is a key factor in the differentiation of mouse B cells into antibody-secreting plasma cells,^37^ while MCP-1, MIP-β, and TNF-α are key factors in antigen-presenting cell activation and migration.^38^

To evaluate the immunogenicity of the vaccine, serum samples were collected at 7, 14, 21, and 28 days post-primary immunization to determine the titers of binding antibodies (Fig. 2c). Binding antibody levels significantly increased after booster immunization, with comparable levels of binding antibodies at days 21 and 28. The titer of anti-E2 antibodies is negative in the placebo group. To further assess the proportion of neutralizing antibodies, we used a strain isolated from Yunnan, China, in 2019 (GenBank: MH670649.1 IOL) and adaptively mutated strains obtained during mouse infection to test neutralizing antibody titers at days 7, 14, 21, and 28 post-primary immunization. Neutralizing antibody levels were barely measurable before booster immunization, with comparable 50% neutralizing titers at days 21 and 28 post-primary immunization. For the adapted strains, the GMTs induced by mCV-1 and mCV-2 at high doses were approximately 291.0 and 220, respectively, while for the original strains, the GMTs reached 668 and 441, respectively, indicating higher levels of neutralizing antibodies against the original strains (Fig. 2d, Supplementary Fig. 2b). One study indicated that CHIKV-neutralizing antibodies are mainly IgG_3_.^39^ Therefore, we assessed the levels of the IgG_3_ subtype in the serum 28 days post-immunization (Supplementary Fig. 10). All vaccine dosage groups elicited a certain level of IgG_3_ subtype antibodies, with mCV-2 demonstrating a slight advantage over mCV-1.

To validate the broad-spectrum protection capability of the vaccines, we conducted pseudovirus neutralization experiments using pseudoviruses from recently reported lineages: GenBank accession numbers AAV43881.1 (West African lineage), AZQ25376.1 (ECSA), UKA87037.1 (IOL), BDD37661.1 (IOL), ADG95944.1 (IOL) and KY435462.1(Aisa). Mice in both vaccine dose groups generated neutralizing antibodies against each pseudovirus strain, with the low dose of both vaccines resulting in GMTs against AAV43881.1 (West African lineage) of approximately 5154 and 6771, which were lower than those against the other pseudovirus strains, while the GMTs against the other four strains at various doses ranged from 3 to 8 × 10^4^ (Fig. 2e, f).

### Both vaccines at each dose induce robust T-cell immune responses, primarily of the Th1 type

**Fig. 3:**
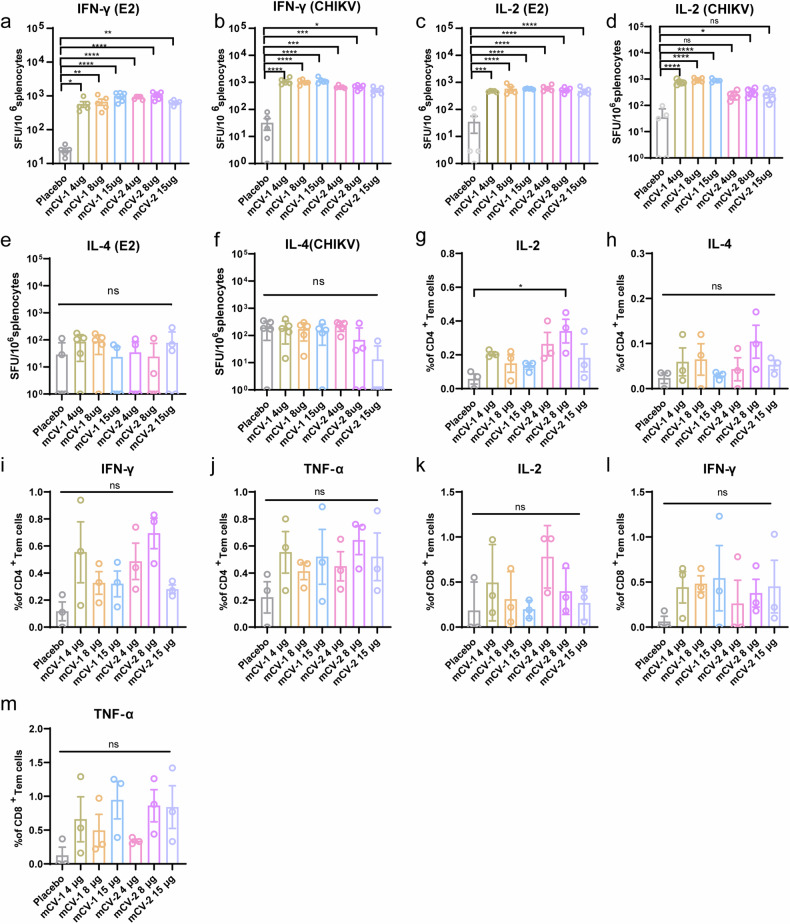
Cellular immune response assessment. **a**–**f** Elispot experiments to enumerate specific spots indicating cytokine secretion by splenocytes stimulated with the E2 protein for IFN-γ (**a**), IL-2 (**c**), and IL-4 (**e**) and stimulated with the inactivated virus for IFN-γ (**b**), IL-2 (**d**), and IL-4 (**f**); **g**–**j** Flow cytometry analysis of the percentages of CD4+ T cells producing IL-2 (**g**), IL-4 (**h**), IFN-γ (**i**), and TNF-α (**j**); **k**–**m** The proportions of CD8+ T cells producing IL-2 (**k**), IFN-γ (**l**), and TNF-α (**m**). The data are presented as the mean ± SEM (*n* = 5 or 3). Statistical analysis was conducted using two-way ANOVA and Tukey’s multiple comparison test for bar graphs; *p < 0.05; **p < 0.01; ***p < 0.001; ****p < 0.0001; ns not significant

To assess the T-cell immune response elicited by the vaccines, splenocytes were collected from immunized mice 28 days post-immunization and subjected to ELISPOT assays to detect cells secreting IFN-γ, IL-2, and IL-4. The E2 protein (IOL) or the inactivated virus (strain MH670649.1) was used as a stimulant. For IL-2 and IFN-γ, dose groups exhibited high numbers of specific spots, with the mCV-2 dose groups stimulated with inactivated virus showing lower levels than the mCV-1 dose groups, possibly due to the absence of antigen targeting the C protein in mCV-2, relatively fewer stimulants than in the mCV-1 group. All other dose groups had significantly greater levels than did the control group (Fig. 3a–d, Supplementary Fig. 2c). For IL-4, regardless of stimulation with the E2 protein or inactivated virus, fewer IL-4-specific spots were generated, but the difference was not significant compared to the number of spots in the placebo group (Fig. 3e, f). High levels of IL-2 and IFN-γ and low levels of IL-4 thus indicate that both vaccines induce a Th1-biased T-cell immune response. To further analyze T-cell immunity and subgroups, flow cytometry was used to determine the proportion of CD4+ and CD8+ T cells that secreted cytokines in response to E2 protein stimulation. Among the CD8+ T cells, cells secreting IL-2, IFN-γ, and TNF-α were detected in all the dose groups, indicating that both vaccines can elicit E2-specific CD8+ T-cell responses (Fig. 3k–m, Supplementary Fig. 3). In immunized mice, high levels of IL-2, IFN-γ, and TNF-α but extremely low levels of IL-4 were detected in CD4+ T cells (Fig. 3g-j), further confirming that both vaccines primarily induce Th1-type T-cell immune responses. It is noteworthy that the flow cytometry results showed no significant difference between the vaccine group and the placebo group, which may be related to individual variability. However, a clear trend induced by vaccination can be observed.

### The mCV-1 and mCV-2 vaccines protect BALB/c mice from challenge with CHIKV

To assess the protective efficacy of the vaccine, mice were challenged with the virus on day 42 after the initial immunization. The adapted strains were utilized for mouse challenge experiments.

**Fig. 4:**
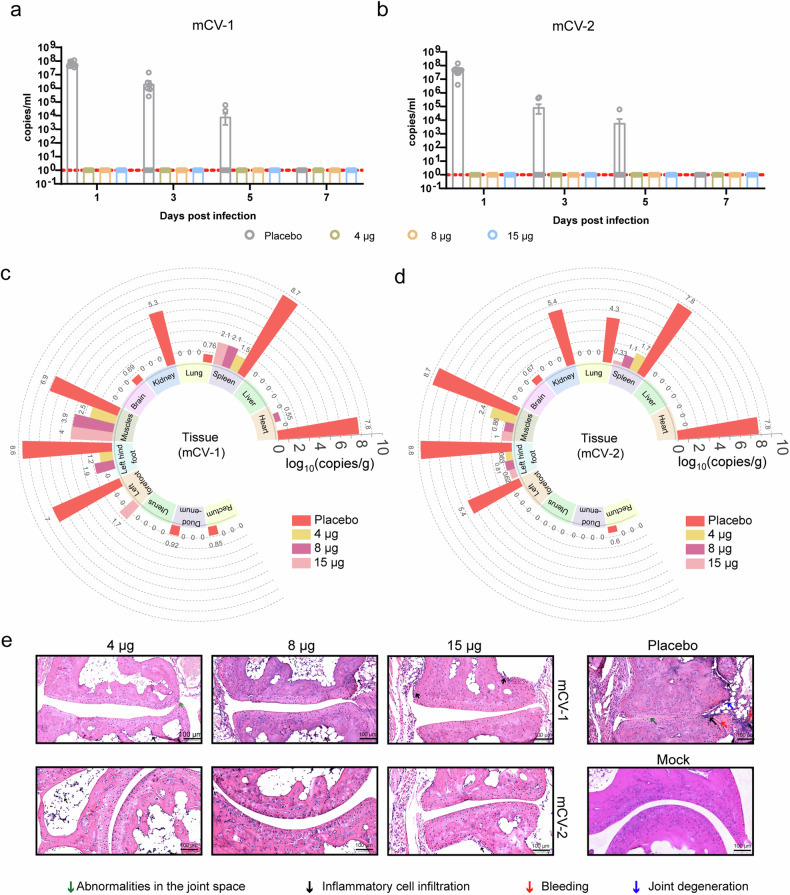
mCV-1 and mCV-2 protect BALB/c mice from CHIKV challenge. **a** and **b** Changes in viremia within 7 days post-immunization with mCV-1 (**a**) and mCV-2 (**b**); Tissue viral loads in mice immunized with mCV-1 (**c**) and mCV-2 (**d**) on day 7 post-challenge; **e** Joint tissue pathological sections and pathological scores, (*n* = 10). The data are shown as the mean ± SEM. Statistical analysis was conducted using two-way ANOVA and Tukey’s multiple comparison test for bar graphs; ***p < 0.001; ****p < 0.0001

To better detect the protective effect of the vaccine, a high virus infection dose was administered, and each mouse was injected subcutaneously at the left and right joints^40^ with a total of 10^6^ plaque-forming units (PFUs) of the virus (the viral load transmitted by infectious Aedes mosquitoes is typically 10^3^ PFU). Subsequently, body weight and joint swelling were monitored on days 1, 3, 5, and 7 post-challenge and blood viral loads were measured via blood sampling via the tail vein. The mice were euthanized on day 7, and the serum, heart, liver, spleen, lung, kidney, brain, infection site muscle, left hindfoot, left forefoot, uterus, duodenum, and rectum were collected for viral load assessment. Both doses of mCV-1 or mCV-2 completely controlled viremia on the first day and resulted in no detectable blood viral load in subsequent tests, whereas the placebo group exhibited a blood viral RNA load reaching 10^7^ copies/ml on the first day (Fig. 4a, b). Due to the self-limiting nature of Chikungunya fever (CHIKVF), the placebo group experienced a continuous decrease in blood viral load in the following days, with viremia disappearing by the 7th day. In the placebo group, the virus was detected in all tissues except the liver and uterus, with varying viral loads measured in the other 10 tissues (Fig. 4c, d), indicating the successful establishment of the adapted strain infection model in BALB/c mice. In the vaccine groups, viral loads were measured in the site of infection (muscle and left hindfoot) and the spleen (an immune organ), with sporadic low-level viral loads observed in the heart and left forefoot in some mice in the low- or medium-dose groups. Compared to the placebo group, the vaccine groups showed a reduction in viral loads in the infected joint and muscle ranging from 7.9 × 10^2^ to 3.98 × 10^8^ copies/g (Fig. 4c, d), indicating good protective efficacy of both vaccines. Due to the lack of obvious joint swelling in the BALB/c mice and measurement error, no significant joint swelling was observed (Supplementary Fig. 4c, d), and there were no differences in body temperature or weight change (Supplementary Fig. 4a, b).

We further measured the neutralizing antibody levels on day 7 post-challenge, which showed a significant increase in neutralizing antibody levels in the vaccine groups compared to pre-challenge levels (Supplementary Fig. 4e, f), while neutralizing antibodies were barely detected in the placebo group. To further confirm the presence of the virus in tissues, we conducted immunofluorescence staining using anti-E1 antibodies, which revealed the presence of viral proteins in the heart, spleen, lung, brain, and muscle in the placebo group (Supplementary Fig. 5). In all vaccine groups, consistent with the viral load results, only a small amount of E1 protein signal was detected in the spleen and muscle.

To further observe pathological damage, we prepared pathological tissue sections from the heart, spleen, lungs, kidneys, brain, and muscles and conducted pathological scoring. CHIKV caused varying degrees of tissue damage to different tissues, with more severe damage observed in the spleen, joints, muscles, and myocardium. In terms of joint damage, the placebo group showed clear abnormalities in the joint space, accompanied by bleeding, joint degeneration, and inflammatory cell infiltration, whereas the vaccine group exhibited significantly reduced damage (Fig. 4e, Supplementary Fig. 6d). The pathological damage to the spleen tissue was primarily characterized by bleeding, inflammatory cell infiltration, germinal center damage, and the disappearance of germinal centers (Supplementary Fig. 6a, d). For muscle, the placebo group showed clear signs of fibrosis, bleeding, and inflammatory cell infiltration, while all doses of the vaccine significantly reduced pathological damage (Supplementary Fig. 6b, d); regarding myocardial tissue, the placebo group exhibited fibrosis, bleeding, intravascular thrombosis, and inflammatory cell infiltration, with the medium and high doses effectively alleviating myocardial tissue pathological damage (Supplementary Fig. 6c, d). In the kidneys, pathological damage was mainly characterized by intravascular thrombosis, bleeding, ductal exudates, and inflammatory cell infiltration (Supplementary Fig. 7a, d). Lung tissue damage was primarily characterized by lung hemorrhage, inflammatory cell infiltration, macrophage distribution, and carbon deposition, thrombus bronchial blockage, and protein exudates (Supplementary Fig. 7c, d). Brain tissue damage was mainly characterized by local hemorrhage, enriched microglia, and neuronal necrosis (Supplementary Fig. 7b, d). In all of the above tissues, all doses of the vaccine significantly reduced the extent of pathological damage.

### mCV-1 and mCV-2 can protect A129 mice from death at low levels of neutralizing antibodies

**Fig. 5:**
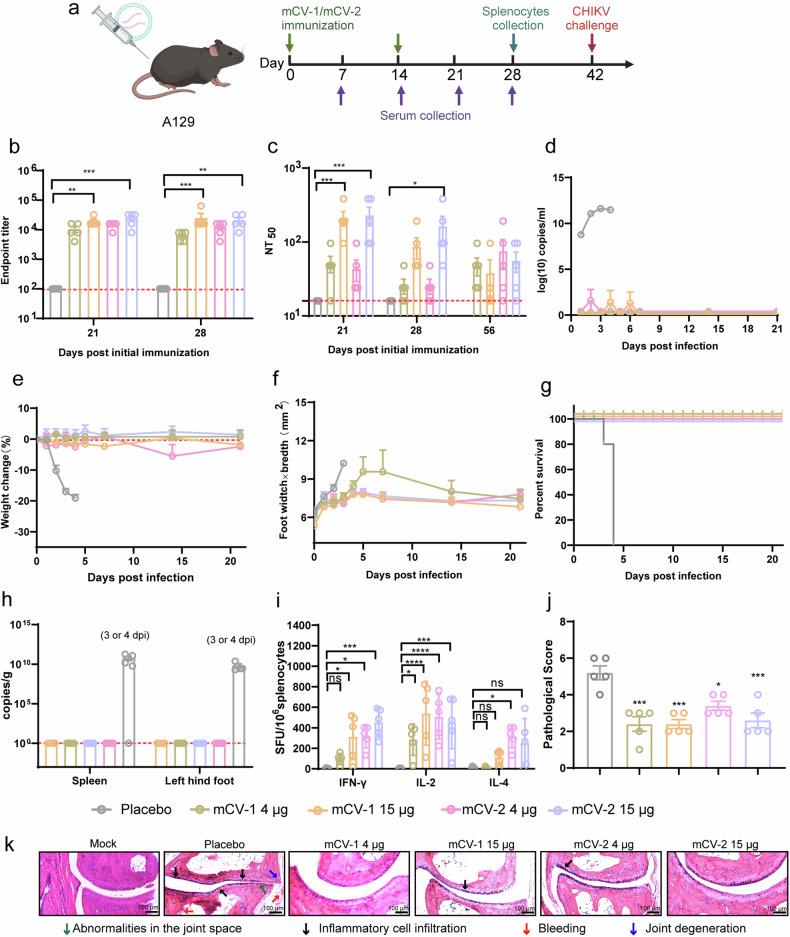
mCV-1 and mCV-2 protect A129 mice from mortality due to CHIKV infection. **a** The vaccination and sample collection timeline, Serum collection, and determination of binding antibody titers and neutralizing antibody levels were performed on days 21 and 28 after initial immunization, followed by challenge experiments on day 42. Body temperature, body weight, and joint changes were monitored daily after the challenge until all mice in the placebo group died, and survival curves were plotted; **b** The titers of binding antibodies at days 21 and 28; **c** Neutralizing antibody titers at 21, 28, and 56 days post-immunization; **d** Variation in viremia within 21 days post-challenge; **e** and **f** Changes in body weight (**e**) and the degree of swelling in the right hind limb joints (**f**) of A129 mice post-challenge; **g** Survival rate of mice post-challenge; **h** Viral loads in the spleen and joint tissues of mice on day 21 post-challenge; **i** Elispots experiments to enumerate specific spots indicating cytokine secretion by splenocytes stimulated with the E2 protein for IFN-γ, IL-2, and IL-4; j-k, Joint tissue pathological sections (**k**) and pathological scores (**j**). Statistical analysis was conducted using one-way ANOVA and Tukey’s multiple comparison tests for bar graphs; *p < 0.05; **p < 0.01; ***p < 0.001; ****p < 0.0001; ns not significant

To further validate the protective efficacy of mCV-1 and mCV-2, we conducted further experiments in A129 mice. mCV-1 and mCV-2 were administered at doses of 15 and 4 μg, respectively. A placebo group with empty LNPs was included, and the same immunization procedure used for BALB/c mice was followed (Fig. 5a). mCV-1 and mCV-2 induced low binding antibody titers in A129 mice (Fig. 5b) and extremely low neutralizing antibody titers (Fig. 5c). In both low-dose vaccine groups, the seroconversion rate was not 100%. Interestingly, after the challenge experiments, all the mice in the vaccine groups, including those without detectable neutralizing antibodies, survived, while all the mice in the placebo group died by day 4 (Fig. 5g). After challenge, there was a rapid decrease in body weight in the placebo group during the first three days, while body weight in the vaccine groups remained within normal ranges (Fig. 5e). Regarding the degree of joint swelling, significant swelling was observed in the placebo group during the first three days, but individual mice in both low-dose vaccine groups exhibited swelling (Fig. 5f). Blood viral loads were measured on days 1–7, 14, and 21 after challenge. Within the first week after the challenge, mild blood viral loads were detected in mice administered mCV-1 at 15 μg and mCV-2 at 4 μg (Fig. 5d), but interestingly, individuals without detectable neutralizing antibodies did not develop viral RNAmia. Neutralizing antibody levels were re-evaluated on day 14 after the challenge, but unlike in BALB/c mice, there was no increase in neutralizing antibody levels after the challenge (Fig. 5c), and there are still individuals who have not produced neutralizing antibodies.

Chronic infection is a major challenge in the treatment of CHIKVF, and viral material may persist for months. Indeed, tissue viral loads were extremely high in the placebo group of A129 mice that died 3–4 days after the challenge. However, in all vaccine dosage groups, no viral RNA was detected in the spleen and joint tissues 21 days post-challenge. (Fig. 5h), demonstrating that the vaccine effectively cleared viral components. The ELIspots experiment revealed that A129 mice also developed a certain level of cellular immunity, predominantly of the Th1 type (Fig. 5i, Supplementary Fig. 8). Pathological scoring of joints and histopathological sections indicated effective control of joint damage across all dose groups in the vaccinated cohort (Fig. 5j, k).

### The usage frequency of IgHV1-4-IgHJ3 and IgHV1-4-IgHJ2 are significantly upregulated in BCR, while the usage frequency of V19 and V20 is significantly upregulated in TCR

To explore the molecular immune mechanisms of the two vaccines, we performed transcriptome, BCR, and TCR sequencing on whole blood samples from mice receiving a medium dose of the vaccines. Untreated mice were used as controls. In terms of transcriptomics, the PCA plot of the samples revealed specific interindividual differences (Supplementary Fig. 9a), and the overall differential expression levels are shown (Supplementary Fig. 9b). Initially, we conducted GO enrichment (|logFC| > 1, *P* < 0.05) and KEGG enrichment (|logFC| > 1, *P* < 0.05) analyses. GO enrichment primarily highlighted the activation and function of natural immune cells, antiviral responses, and complement-related pathways (Supplementary Fig. 9c, d). Both vaccine groups showed KEGG enrichment in Fc epsilonRI, B-cell receptor signaling, NK cell-mediated phagocytosis, and Fc receptor-mediated phagocytosis (Supplementary Fig. 9e, f). Therefore, the immune mechanism post-vaccination may involve the action of Fc segment receptors.^41^

**Fig. 6:**
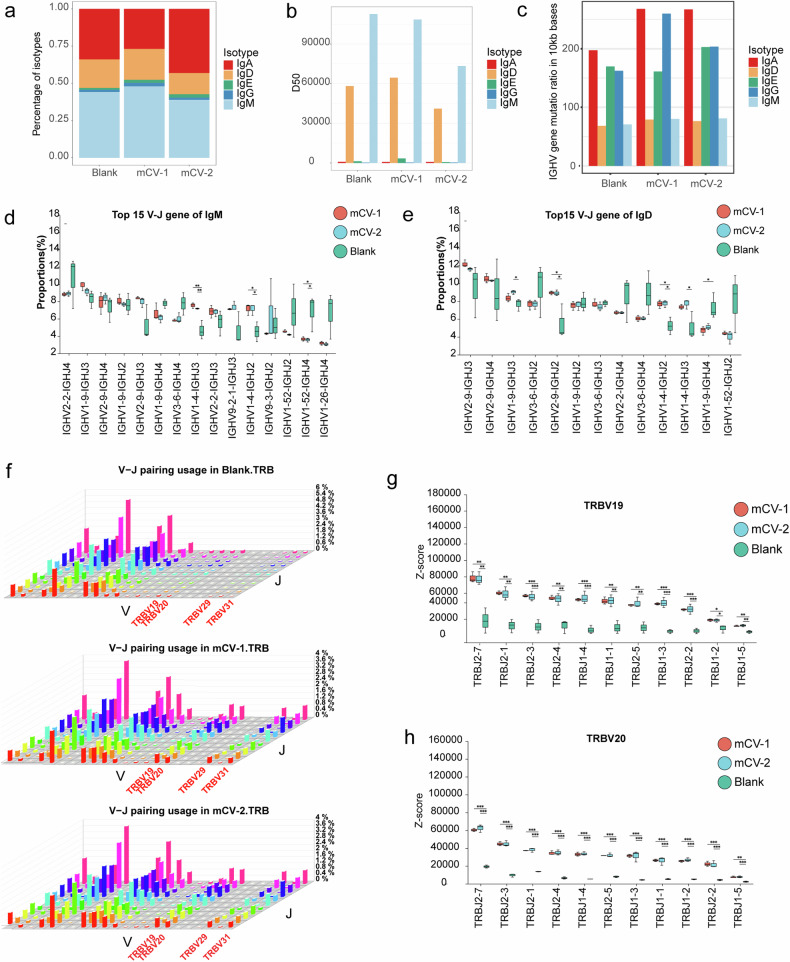
BCR and TCR immune repertoire analysis in BALB/c. **a** Abundance of immunoglobulin types in the three groups. **b** Statistical analysis of immunoglobulin diversity; **c** Analysis of the gene mutation of IgM, IgD, IgA and IgG; **d** and **e** Significant differences in the abundance of the 15 most abundant V-J gene combinations for BCRs for IgM and IgD; **f** VDJ matrix of TCR sequences for the three samples; **g** and **h** Significant differences in the V-J gene combinations containing V19 (**g**) and V20 (**h**) for TCRs. Statistical analysis was conducted using one-way ANOVA and Tukey’s multiple comparison tests for bar graphs; *p < 0.05; **p < 0.01; ***p < 0.001; ****p < 0.0001; ns not significant

Regarding BCR sequencing, the abundance of different immunoglobulin types showed that IgM, IgA, and IgD were the most abundant in all three samples, with IgM and IgD being the most diverse (Fig. 6a, b). IgD diversity increased with mCV-2, while both IgD and IgM decreased with mCV-2. This result suggests a potential post-immunization contraction in antibody diversity, concentrating the production of specific antibodies. Somatic hypermutation is a mechanism diversifying the BCR repertoire during the germinal center reaction. Mutation rate analysis reveals that IgM and IgD, which exhibit the highest diversity, do not necessarily have high mutation rates (Fig. 6c), indicating that mutation is not the primary driver of diversity. Post-immunization, there is a notable increase in mutation rates for IgA and IgG. We further evaluated the utilization of V-J genes in the heavy chains of IgD and IgM in the two vaccine groups and the control group. For robust function post-immunization, V-J gene combinations need to reach high abundances. We selected the 15 most abundant combinations for further significant difference analysis and found that the abundances of the IgHV1-4-IgHJ3 and IgHV1-4-IgHJ2 combinations in the two vaccine groups were significantly greater than those in the control group (Fig. 6d, e), possibly because of conserved broad-spectrum epitopes. Similarly, analysis of the V-J gene usage frequency in TCRs (Fig. 6f) revealed a significant increase in the TCR combinations of V19 and V20 (Fig. 6g, h, Supplementary Fig. 9g, h), indicating that during the initial immune response phase, antigen capture and presentation may occur through the use of V19 and V20 TCRs, along with humoral immunity.

### The mCV-1 and mCV-2 vaccines protect rhesus macaques from challenge with CHIKV

**Fig. 7:**
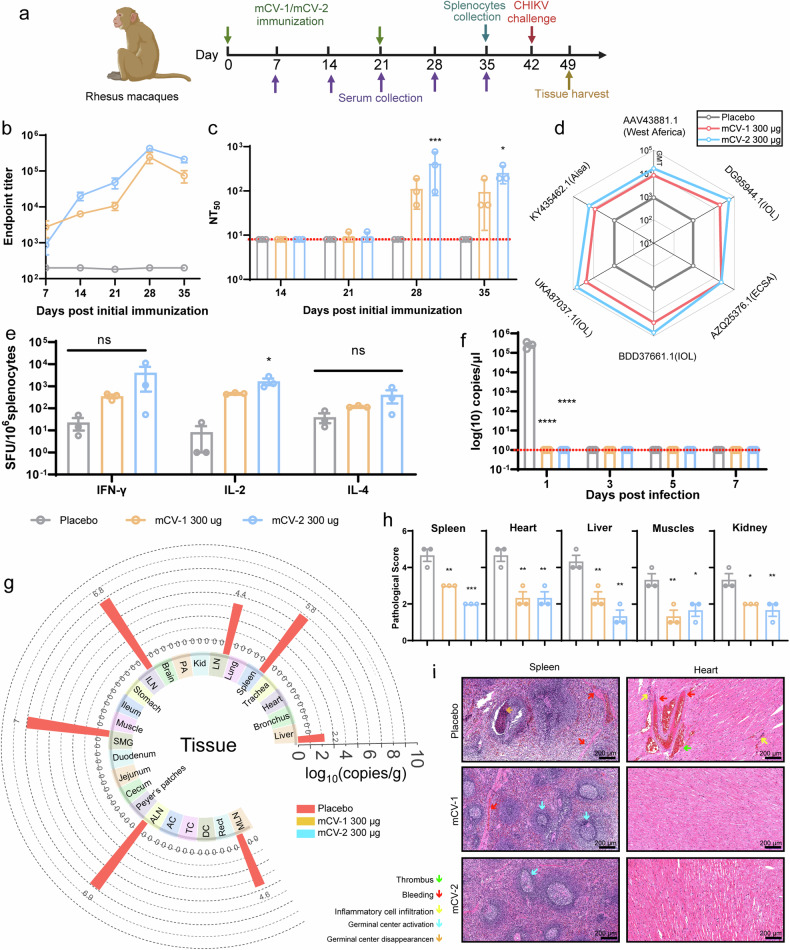
mCV-1 and mCV-2 protect rhesus macaques from CHIKV challenge. **a** The immunization schedule for rhesus macaques is depicted in the flowchart, with vaccinations administered on Day 0 and Day 21, at a dosage of 300 μg per animal, using physiological saline as the placebo group. Serum samples were collected on Days 7, 14, 21, 28, and 35 post-primary immunization, followed by a challenging experiment on Day 42 (*n* = 3); **b** and **c** The titers of binding antibodies (**b**) and neutralizing antibodies against adapted strains (**c**) at different time points; **d** GMTs of pseudovirus neutralizing antibodies against various strains at 35-day post-immunization for mCV-1 and mCV-2; **e** Elispot experiments to enumerate specific spots indicating cytokine secretion by splenocytes stimulated with the E2 protein for IFN-γ, IL-2, and IL-4; **f** Changes in viremia within 7 days post-immunization with mCV-1 and mCV-2; **g** Tissue viral loads in rhesus macaques immunized with mCV-1, mCV-2 or physiological saline on day 7 post-challenge, LN: Hilar lymph nodes, Kid: Kidney, PA: Pancreas, ILN: Inguinal lymph nodes, MLN: mesenteric lymph nodes, SMG: submandibular lymph node; **h** and **i** pathological scores (**h**) and pathological sections (**i**). The data are presented as The mean ± SEM (*n* = 3), and each symbol represents a mouse. Statistical analysis was conducted using one-way ANOVA and Tukey’s multiple comparison tests for bar graphs; ***p < 0.001; ****p < 0.0001; ns not significant

To further evaluate the protective efficacy of these two vaccines, we conducted an immunization-challenge experiment in rhesus macaques. The experimental procedure is depicted (Fig. 7a). ELISA results indicated a significant increase in binding antibody levels after the booster immunization (Fig. 7b). Live virus neutralization assay results showed that neutralizing antibodies were not detected before the booster immunization (Fig. 7c, Supplementary Fig. 11a), but were detected at certain levels after the booster immunization, similar to the mouse experiments, with neutralizing antibody levels against the adapted strain being lower than those against the prototype strain (MH670649.1). Pseudovirus neutralization assay results from four lineages demonstrated that both mCV-1 and mCV-2 exhibit broad-spectrum efficacy against different lineages (Fig. 7d). In terms of cellular immunity, ELISpots assays revealed that both vaccines can elicit a certain level of cellular immunity in rhesus macaques (Fig. 7e, Supplementary Fig. 11b).

Post-challenge, body temperature, and weight were not significantly affected (Supplementary Fig. 11c, d). The control group exhibited noticeable viremia on the first-day post-challenge (Fig. 7f), whereas the vaccine groups did not show any signs of viremia. In the detection of viral RNA in various tissues, the control group had detectable viral RNA loads in lymph nodes, spleen, and other immune organs such as the liver, while the vaccine groups did not (Fig. 7g). The control group showed more severe damage in the spleen, myocardial tissue, liver, leg muscles, and kidney tissues, characterized by hemorrhage and thrombosis, inflammatory cell infiltration. The spleen exhibited active and disappearing germinal centers; muscle and myocardial tissues showed myofibrosis (Fig. 7h, i, Supplementary Fig. 11e).

## Discussion

As the survival range of Aedes mosquitoes expands and climate change progresses, the threat of epidemics caused by alphaviruses,^6^ such as CHIKV, is becoming increasingly severe. Our study presents two mRNA vaccines with potential for cross-lineage protection, providing a comprehensive evaluation of immunogenicity and efficacy in BALB/c mice and further validating their protection effectiveness in the A129 mouse model and rhesus macaque model.

Currently, only one live attenuated vaccine (VLA1553) has been approved for market use. Compared to VLA1553, the levels of E2-specific binding antibodies induced by mCV-1 and mCV-2 range from 10^4^ to 10^5^, similar to those elicited by a single dose of VLA1553.^42^ In terms of neutralizing antibody levels, a single dose of VLA1553 generates neutralizing antibody titers between 10^2^ and 10^4^, while neutralizing antibody titers after two immunizations with mCV-1 and mCV-2 range from 10^2^ to 10^3^, with an average neutralizing antibody titer >10^3^ observed post-challenge. Regarding protective efficacy, both vaccines successfully controlled viremia in BALB/c or C57BL/6 mice. Additionally, ChAdOx1 Chik employs a similar conservative antigen design,^43^ and preclinical studies have shown it can induce a strong cellular immune response, with neutralizing antibody levels increasing and stabilizing over time. Phase I clinical trials detected neutralizing antibodies against four lineages of CHIKV.^44^

mCV-1 and mCV-2, after immunization in BALB/c mice and A129 mice, induced the production of high levels of binding antibodies. However, compared to the extremely high levels of neutralizing antibodies produced in response to immunization with some attenuated vaccines,^45,46^ the proportions of neutralizing antibodies generated in response to mCV-1 and mCV-2 were not very high. Previous studies have indicated that the highly conserved E1 protein induces the generation of nonneutralizing antibodies and represents a potential target for broad-spectrum vaccine design.^25,26^ These nonneutralizing antibodies could be a crucial component conferring broad-spectrum protection. Despite the modest levels of neutralizing antibodies, particularly in A129 mice, complete control of viremia was still achieved, and protection against mortality in A129 mice was observed in CHIKV infection. mCV-1 and mCV-2 also induced strong cellular immune responses, primarily Th1-type CD4+ and CD8+ T cells. Excessive activation of Th2 cells after vaccination can lead to Th2-related vaccine-associated enhanced diseases (VAEDs), but no significant increase in IL-4 or occurrence of VAEDs was observed after mCV-1 and mCV-2 immunization. Following the challenge, significant increases in viral loads and alleviation of pathological damage were observed in 11 extrarticular tissues of BALB/c mice, and chronic infection was not observed in A129 mice. For mCV-1 and mCV-2, studies have shown that after vaccine immunization, some CD8+ T cells targeting the C protein can be generated,^37^ but in terms of overall protective efficacy, mCV-2 without the C protein has an effect equal to that of mCV-1, and the absence of the C protein also reduces the complexity of the antigen and reduce the proportion of non-neutralizing antibodies.

Consensus synthetic sequence approach have been applied in other fields, such as virus detection.^35^ CHIKV consists of four genotypes, and there is a high degree of conservation among these genotypes. Vaccines prepared based on one strain may exhibit certain cross-protection against other genotypes, but studies have shown differences in infection and immune mechanisms between different lineages, as well as significant differences in serum antibody levels.^24^ Pseudovirus neutralization experiments have shown that pseudovirus neutralizing antibodies against the West African lineage are approximately 7 times lower than those against the Indian Ocean lineage, and there is a one amino acid difference in E2 protein against the two strains of live virus, but there is a significant difference in their neutralizing antibody titers. Results from the alphavirus neutralization assay showed that mCV-1 and mCV-2 are more suitable for the Indian Ocean lineage strains, which have a wide range of transmission and stronger mutation capabilities, and they are more adaptable to the emergence of new IOL mutant strains.

Furthermore, according to the evaluation of the vaccine in A129 mice, the immunogenicity of both vaccines was limited, Some individuals did not develop neutralizing antibodies, yet they still demonstrated good protection post-challenge. Previous studies have extensively investigated the role of neutralizing antibody levels in viral clearance.^42,47,48^ However, the titers of neutralizing antibodies required for effective function do not need to be very high. Studies have shown that passive transfer of immune serum can protect A129 mice from mortality, and there is a significant negative correlation between binding IgG antibody levels and footpad swelling and viremia in C57BL/6 mice.^42,45,49^ Research has indicated that the minimum IgG titer required to prevent viremia and footpad swelling in the C57BL/6 model is 10^4^^45^. However, in A129 mice, seemingly lower IgG titers also provide effective protection. For viral clearance, the use of monoclonal antibodies has further confirmed the decisive role of neutralizing antibodies in viral elimination.^50^ There is evidence suggesting that efficient clearance and alleviation of joint swelling by CHIKV indicate the involvement of pathways mediated by antibody-Fc-FcγR-guided monocytes.^51^ Our transcriptome analysis of BALB/c mice also revealed enrichment in pathways related to ADCP and Fc receptors. Numerous studies have reported results similar to ours, showing that low levels of neutralizing antibodies can achieve complete protection,^52,53^ suggesting that nonneutralizing antibodies may also contribute to viral clearance through antibody-Fc effector functions.

Additionally, non-structural proteins play a significant role in immunity and are potential targets for vaccine development.^54^ Research has predicted CD8+ T cell epitopes within these non-structural proteins, which can elicit a certain level of cellular immunity. This response is crucial for viral clearance and the protective efficacy of vaccines.^55^

Given the significant differences between mice and humans in genetic background and immune response, evaluating vaccines in mouse models does not adequately reflect the true efficacy of the vaccines. Therefore, using animal models that have been validated to simulate human CHIKV infection is crucial for the evaluation of CHIKV vaccines. There are certain differences in immunogenicity and challenge results between rhesus macaques and mice. The levels of neutralizing antibodies in rhesus macaques are comparable to those in convalescent patients, while the cellular immune response is weaker than that in the mouse model. It is possible that in the rhesus macaque model, neutralizing antibodies play a more significant role, and there are also differences in the sites of viral infection post-challenge compared to the mouse model. Additionally, vaccine mCV-2 demonstrated higher levels of neutralizing antibodies and cellular immunity compared to mCV-1, likely due to the higher content of the main antigens E2/E1 in mCV-2 at the same dosage. However, in terms of protective efficacy, both vaccines showed good protective effects.

Compared to previous vaccines, the binding antibody levels elicited in non-human primates are comparable, while the levels of neutralizing antibodies are lower than those of the attenuated live CHIKV vaccine (Δ5nsP3),^46,56^ DNA-launched replicon vaccine, and recombinant modified vaccinia Ankara virus.^56^ This may be due to the design of conserved sequences that enhance cross-protection between lineages while reducing specificity for particular strains. However, both of these vaccines provide the same level of challenge protection as previous vaccines, with no viremia detected. Moreover, no virus-related components were detected in the tissues of the mRNA vaccine group after euthanasia. Variations in experimental conditions and the use of different strains can also lead to differences in experimental outcomes. However, the protective efficacy has been robustly demonstrated. The rhesus macaque experiments further highlight the potential of mCV-1 and mCV-2 as candidate vaccines for clinical application.

Based on the favorable protective effects described above, we consider both mCV-1 and mCV-2 to be potential candidate vaccines for further evaluation in subsequent phase I studies.

### Limitations of the study

The challenge experiment in this study was conducted with only a single strain of live virus. Second, the three selected doses in this study did not exhibit significant dose dependency. Furthermore, the adaptive immune response mechanisms have not been thoroughly validated, which will require further research and evaluation in the future.

## Materials and methods

### Animals and ethics statement

BALB/c mice at 6–8 weeks of age and rhesus macaques aged 4–8 years were obtained from the Institute of Medical Biology, Chinese Academy of Medical Sciences, while A129 mice at 6–8 weeks of age were purchased from the National Institutes for Food and Drug Control. All animals were kept in a specific pathogen-free (SPF) environment. The animal experiments were approved by the Animal Experiment Ethics Committee of the Institute of Medical Biology, Chinese Academy of Medical Sciences (DWSP202405001, DWSP20240309), and conducted in accordance with the Guidelines for the Care and Use of Laboratory Animals by the National Institutes of Health. All experiments involving the infection of CHIKV were carried out in a biosafety level 3 (ABSL3) facility at the Institute of Medical Biology, Chinese Academy of Medical Sciences.

### Cells and viruses

293T/17 cells were purchased from Wuhan Pricella Biotechnology, while Vero cells were sourced from our laboratory stocks. Both types of cells were cultured in Dulbecco’s Modified Eagle Medium (DMEM, #8123187) containing 10% fetal bovine serum (FBS, 2440086), with the addition of 1% penicillin–streptomycin, at 37 °C in a 5% CO_2_ environment. The viral strain MH670649.1 was derived from an imported case in Ruili, China, in 2019, and was isolated by the Yunnan Institute of Parasitic Diseases. The adapted strain was obtained through adaptive mutation during our mouse infection experiments. CHIKVs were passaged in Vero cells, and the virus stock was aliquoted and titrated to plaque-forming units per milliliter (PFU/ml) in Vero cells using a plaque assay. All experiments involving infectious agents were conducted in the BSL-3 facilities at IMBCAMS.

### mRNA synthesis

Initially, plasmids were linearized using the BsaI enzyme, followed by precipitation of the DNA template with 70% isopropanol, washing with 70% ethanol, and resuspension in DEPC water. In vitro transcription (IVT) was performed using a commercial kit (NEB#E2080S) according to the manufacturer’s instructions, with N1-Methylpseudouridine-5-Triphosphate replacing unmodified UTP. The resulting mRNA was precipitated with a LiCl solution and washed with 70% ethanol. Agarose gel electrophoresis was conducted to assess the integrity and purity of the mRNA.

### LNP encapsulation of mRNA

First, SM102, PEG2000-DMG, DSPC, and cholesterol were mixed in molar ratios of 50%, 1.5%, 10%, and 38.5%, respectively, to prepare a lipid–ethanol solution with a total lipid concentration of 16 mM. The mRNA was diluted to 108 ng/μl in citrate buffer at pH = 4 and mixed with the lipid solution using a microfluidic device (Fluidiclab) at ethanol: buffer flow rate ratio of 5 (ml/min):15 (ml/min) to prepare mRNA-LNP. The formulations were diluted 10 times with citrate buffer, ultrafiltered, and the citrate buffer was replaced with Tris–HCl buffer. The RNA concentration and encapsulation rate were measured using the Quant-it RiboGreen RNA assay kit (Invitrogen, #R11490), and the size of the mRNA-LNP particles was measured using dynamic light scattering with a Zetasizer Pro (Malvern).

### mRNA in vitro expression

Vero cells or 293T/17 cells were cultured in 24-well plates until reaching 80% confluency. The culture medium was then replaced with Opti-MEM (gibco, #31985070), and 1 μg of mRNA encapsulated in LNPs was added to the cells. Alternatively, commercially available transfection kits (Mirus, MIR2250) were used to transfect naked mRNA, with cells without mRNA addition serving as negative controls. mCV-1, mCV-2, and the mock control were each performed in triplicate. After transfection for 6 h, the medium was replaced with a complete culture medium. After 24 h, the supernatant was discarded, and the cells were washed with PBS before being lysed with RIPA lysis buffer. Western blot experiments were performed using antibodies against E1(GeneTex #GTX135187), E2 (Alpha Diagnostic International, #CHIKE21-A), and C (Gene Tex, #GTX135183) proteins, with β-actin serving as a reference.

### Antibody endpoint titer measurement with ELISA

E2 protein (Sino Biological, #40440-V08B, 1 μg/ml, 100 μl/well) was coated in a 96-well plate (442404, Thermo Fisher Scientific) overnight at 4 °C. After washing three times with PBST (0.05% Tween-20 in PBS), the plate was blocked with 2% BSA in PBS at 37 °C for 1 h. Serum samples were diluted in dilution buffer (0.05% BSA in PBST) starting from 1:200, followed by serial two-fold dilutions and incubated at 37 °C for 1 h. After washing three times with PBST, goat anti-mouse IgG (A-10668, Invitrogen) or rabbit anti-monkey IgG (A2054-1ML, sigma, dilution:1:45,000) conjugated with horseradish peroxidase was diluted 1:30,000 in sample diluent and 100 μl was added to each well. The plate was then incubated again at 37 °C for 1 h followed by three washes with PBST. Subsequently, 100 μl of TMB substrate (Thermo Fisher Science) was added to each well and incubated at room temperature for 15 min. The reaction was stopped using a stop solution (SolarBio, C1058). Absorbance was measured at 450 and 630 nm. Wells with no serum served as blank controls, and the threshold was set at 2.1 times the positive result control. Positive results were interpreted as OD_450–630nm_ > 0.1. IgG endpoint titers were defined as the dilution factor.

### Detection of inflammatory factors in serum

Serum samples from non-immunized mice were collected as 0-hour samples, followed by serum collection at 5 and 24 h post-immunization. The Bio-Plex Pro kit (BioRAD, #64521678) was utilized and assays were conducted on the Bio-Plex system (Bio-Rad) following the manufacturer’s protocol.

### Live CHIKV virus neutralization assay

To determine the serum-neutralizing antibodies against the live CHIKV virus, we performed the authentic virus neutralization assay. Mouse serum was inactivated at 56 °C for 30 min, then serially diluted two-fold starting from 1:16 to 1:2048 with DMEM medium in 96-well plates for micro-neutralization assay. Each well contains 50 μl of each diluted serum sample. Viruses were diluted to 2000 PFU/ml with DMEM, and 50 μl of the diluted virus was added to each well containing an equal volume of diluted serum sample, followed by incubation at 37 °C for 1 h. Then 100 μl of Vero cells (1.5 × 10^4^ cells/well) were added to 96-well plates and incubated for 5–7 days. The cytopathogenic effects (CPE) were observed by microscope to evaluate the neutralizing ability of the serum sample. The Karber method was used to calculate the neutralizing antibodies titers, which was the highest serum dilution resulting in 50% neutralization inhibition. For example, the sample was serially diluted two-fold, and 2 parallel holes were set for each dilution gradient. To quickly calculate neutralizing antibody titers, If CPE occurs in one well and no CPE occurs in the other well at a certain dilution, the reciprocal of the dilution was used as the titer of the serum-neutralizing antibody. If there was no CPE in a certain dilution test hole, the average of the reciprocal dilution and the reciprocal dilution of the next dilution was used as the titer of the serum-neutralizing antibody.

### Pseudovirus-based neutralization assay

A total of 5 CHIKV pseudoviruses were employed to evaluate the overall neutralizing antibody titers in the serum of immunized mice. All pseudoviruses were detected with assistance from the research group of Youchun Wang. The serum to be tested was heat-inactivated at 56 °C for 30 min. Starting with an initial dilution of 1:90 (rhesus macaque, 1:900), the serum was serially diluted threefold in a 96-well plate. The pseudovirus was incubated with serially diluted serum samples from mice immunized with vaccine at 37 °C for 1 h, and then mixed with 293T cells in a 96-well plate, followed by incubation for 48 h. The infectivity of pHIV-CHIKV-Fluc was determined by measuring bioluminescence. The 50% inhibitory dilution (ID50) is defined as the neutralizing antibody titer of the serum. The dilution range is 90–1,968,300.

### ELISPOTS assay

The ELISPOTS assay was conducted according to the manufacturer’s instructions (Mabtech). In brief, mouse spleens were harvested and PBMCs were isolated following the instructions of the kit (Solabio, #P8860). Stimulation of cells was performed using E2 protein (2 μg/10^6^ cells) or inactivated virus (10^5^/well); unstimulated cells were as a negative control for every sample and cells stimulated by PHA was as a positive control. After 24 h, followed by equilibration, washing, and stimulation of cells in a 96-well plate (3321-4APT-10, 3441-4APW-10, 3311-4APW-2, Mabtech) according to the manufacturer’s instructions, and cell counting and photography were performed on MABTECH IRIS^TM^.

### Quantification of viral load in mice

The viral RNA load in the blood and tissues of challenged mice was detected by RT–qPCR. Blood samples were mixed with Trizol at a ratio of 1:3, while tissue samples weighing 100–200 mg were added to 800 μl Trizol and thoroughly homogenized for inactivation. RNA was extracted from 200 μl Trizol samples using the UPure Virus RNA Plus Kit (Cat# M2006P-A96, Biokeystone, China), and the extracted nucleic acids were used for RT-qPCR detection. RT-qPCR detection was performed using TaqMan Fast Virus 1-Step Master Mix (Cat# 4444432, Thermo Fisher Scientific, USA) on a CFX384 Touch Real-Time PCR Detection System (Bio-Rad, USA). Primers and probes targeting the viral structural protein were synthesized as follows: CHIKV-F: 5’-AAGCTCCGCGTCCTTTACCAAG-3’, CHIKV-R: 5’-CCAAATTGTCCTGGTCTTCCT-3’, CHIKV-Probe: 5’-FAM-CCAATGTCTTCAGCCTGGACACCTTT-TAMRA-3’. Plasmids containing the target were used as standard samples after gradient dilution. One-step RT-PCR was carried out under the following conditions: 2 min at 25 °C, 15 min at 50 °C, and 2 min at 95 °C, followed by 40 cycles of 5 s at 95 °C and 31 s at 60 °C.

### Histopathology

The tissues were fixed in 4% paraformaldehyde solution for 72 h, embedded in paraffin, and sectioned at 5 μm thickness for hematoxylin and eosin (H&E) staining. The sections were scanned by 3DHISTECH. The HE-stained sections were evaluated by experienced pathologists using the CaseViewer provided by the manufacturer for scoring.

## Supplementary information

Revised 4 supplementary information

## Data Availability

All study data are included in the article and/or supplementary information. The raw data of RNA/BCR/TCR sequencing in this study is available at SRA: PRJNA1144660; PRJNA1154416.

## References

[1] Levi, L. I. & Vignuzzi, M. Arthritogenic alphaviruses: a worldwide emerging threat? *Microorganisms* **7**, 133 (2019). 31091828 10.3390/microorganisms7050133 PMC6560413

[2] Nsoesie, E. O. et al. Global distribution and environmental suitability for chikungunya virus, 1952 to 2015. *Eurosurveillance* **21**, 30234 (2016). 10.2807/1560-7917.ES.2016.21.20.30234 PMC4902126 27239817

[3] Suhrbier, A. Rheumatic manifestations of chikungunya: emerging concepts and interventions. *Nat. Rev. Rheumatol.* **15**, 597–611 (2019). 31481759 10.1038/s41584-019-0276-9

[4] Chuong, C. et al. Enhanced attenuation of chikungunya vaccines expressing antiviral cytokines. *npj Vaccines* **9**, 59 (2024). 38472211 10.1038/s41541-024-00843-x PMC10933427

[5] Bartholomeeusen, K. et al. Chikungunya fever. *Nat. Rev. Dis. Primers.* **9**, 17 (2023). 37024497 10.1038/s41572-023-00429-2 PMC11126297

[6] Kramer, I. M. et al. The ecophysiological plasticity of Aedes aegypti and Aedes albopictus concerning overwintering in cooler ecoregions is driven by local climate and acclimation capacity. *Sci. Total Environ.* **778**, 146128 (2021). 34030376 10.1016/j.scitotenv.2021.146128

[7] Tjaden, N. B. et al. Modelling the effects of global climate change on Chikungunya transmission in the 21st century. *Sci. Rep.* **7**, 3813 (2017). 28630444 10.1038/s41598-017-03566-3 PMC5476675

[8] Srikirin, P. et al. Prevalence, risk factors, and prognosis of liver involvement in adult patients with chikungunya in Thailand. *Am. J. Trop. Med. Hyg.* **107**, 1107 (2022). 36252802 10.4269/ajtmh.22-0339 PMC9709004

[9] Do Nascimento Costa, D. M. et al. Chikungunya virus as a trigger for different renal disorders: an exploratory study. *J. Nephrol.* **35**, 1437–1447 (2022). 35119686 10.1007/s40620-022-01256-6

[10] Schmidt, C. & Schnierle, B. S. Chikungunya vaccine candidates: current landscape and future prospects. *Drug. Des. Dev. Ther.* **16**, 3663–3673 (2022). 10.2147/DDDT.S366112 PMC9580835 36277603

[11] Larrieu, S. et al. Factors associated with persistence of arthralgia among Chikungunya virus-infected travellers: report of 42 French cases. *J. Clin. Virol.* **47**, 85–88 (2010). 20004145 10.1016/j.jcv.2009.11.014

[12] Borgherini, G. et al. Persistent arthralgia associated with chikungunya virus: a study of 88 adult patients on Reunion Island. *Clin. Infect. Dis.* **47**, 469–475 (2008). 18611153 10.1086/590003

[13] Sissoko, D. et al. Post-epidemic Chikungunya disease on Reunion Island: course of rheumatic manifestations and associated factors over a 15-month period. *PLoS Negl. Trop. Dis.* **3**, e389 (2009). 19274071 10.1371/journal.pntd.0000389 PMC2647734

[14] Soumahoro, M.-K. et al. Impact of Chikungunya virus infection on health status and quality of life: a retrospective cohort study. *PLoS ONE* **4**, e7800 (2009). 19911058 10.1371/journal.pone.0007800 PMC2771894

[15] Soumahoro, M.-K. et al. The Chikungunya epidemic on La Reunion Island in 2005–2006: a cost-of-illness study. *PLoS Negl. Trop. Dis.* **5**, e1197 (2011). 21695162 10.1371/journal.pntd.0001197 PMC3114750

[16] Alvis-Zakzuk, N. J. et al. Economic costs of chikungunya virus in Colombia. *Value Health Reg. Issues* **17**, 32–37 (2018). 29627722 10.1016/j.vhri.2018.01.004

[17] Costa, L. B. et al. Epidemiology and economic burden of chikungunya: a systematic literature review. *J. Travel. Med.* **8**, 301 (2023). 10.3390/tropicalmed8060301 PMC10302198 37368719

[18] Rajapakse, S., Rodrigo, C. & Rajapakse, A. Atypical manifestations of chikungunya infection. *Trans. R. Soc. Trop. Med. Hyg.* **104**, 89–96 (2010). 19716149 10.1016/j.trstmh.2009.07.031

[19] De Lima Cavalcanti, T. Y. V., Pereira, M. R., de Paula, S. O. & Franca, R. F. D. O. A review on chikungunya virus epidemiology, pathogenesis and current vaccine development. *Viruses* **14**, 969 (2022). 35632709 10.3390/v14050969 PMC9147731

[20] Phadungsombat, J. et al. Spread of a novel Indian Ocean Lineage carrying E1-K211e/E2-V264A of chikungunya virus east/central/South African genotype across the Indian subcontinent, Southeast Asia, and eastern Africa. *Microorganisms* **10**, 354 (2022). 35208808 10.3390/microorganisms10020354 PMC8878743

[21] Presti, A. L., Cella, E., Angeletti, S. & Ciccozzi, M. Molecular epidemiology, evolution and phylogeny of Chikungunya virus: an updating review. *Infect. Genet. Evol.* **41**, 270–278 (2016). 27085290 10.1016/j.meegid.2016.04.006

[22] Song, H. et al. Molecular basis of arthritogenic alphavirus receptor MXRA8 binding to chikungunya virus envelope protein. *Cell* **177**, 1714–1724e1712 (2019). 31080063 10.1016/j.cell.2019.04.008

[23] Li, L., Jose, J., Xiang, Y., Kuhn, R. J. & Rossmann, M. G. Structural changes of envelope proteins during alphavirus fusion. *Nature* **468**, 705–708 (2010). 21124457 10.1038/nature09546 PMC3057476

[24] Chua, C.-L., Sam, I.-C., Merits, A. & Chan, Y.-F. Antigenic variation of East/Central/South African and Asian chikungunya virus genotypes in neutralization by immune sera. *PLoS Negl. Trop. Dis.* **10**, e0004960 (2016). 27571254 10.1371/journal.pntd.0004960 PMC5003353

[25] Williamson, L. E. et al. Therapeutic alphavirus cross-reactive E1 human antibodies inhibit viral egress. *Cell* **184**, 4430–4446e4422 (2021). 34416147 10.1016/j.cell.2021.07.033 PMC8418820

[26] Kim, A. S. & Diamond, M. S. A molecular understanding of alphavirus entry and antibody protection. *Nat. Rev. Microbiol.* **21**, 396–407 (2023). 36474012 10.1038/s41579-022-00825-7 PMC9734810

[27] Staikowsky, F. et al. Retrospective survey of Chikungunya disease in Reunion Island hospital staff. *Epidemiol. Infect.* **136**, 196–206 (2008). 17433130 10.1017/S0950268807008424 PMC2870803

[28] Ly, H. Vol. 15 2301573 (Taylor & Francis, 2024).

[29] McMahon, R. et al. A randomized, double-blinded phase 3 study to demonstrate lot-to-lot consistency and to confirm immunogenicity and safety of the live-attenuated chikungunya virus vaccine candidate VLA1553 in healthy adults. *J. Travel. Med.* **31**, taad156 (2024). 38091981 10.1093/jtm/taad156 PMC10911060

[30] Ye, Z. et al. The mRNA Vaccine Revolution: COVID-19 has launched the future of vaccinology. *ACS Nano* **17**, 15231–15253 (2023). 37535899 10.1021/acsnano.2c12584

[31] Barbier, A. J., Jiang, A. Y., Zhang, P., Wooster, R. & Anderson, D. G. The clinical progress of mRNA vaccines and immunotherapies. *Nat. Biotechnol.* **40**, 840–854 (2022). 35534554 10.1038/s41587-022-01294-2

[32] Zhang, G., Tang, T., Chen, Y., Huang, X. & Liang, T. mRNA vaccines in disease prevention and treatment. *Signal. Transduct. Target. Ther.* **8**, 365 (2023). 37726283 10.1038/s41392-023-01579-1 PMC10509165

[33] Rzymski, P., Szuster‐Ciesielska, A., Dzieciątkowski, T., Gwenzi, W. & Fal, A. mRNA vaccines: the future of prevention of viral infections? *J. Med. Virol.* **95**, e28572 (2023). 36762592 10.1002/jmv.28572

[34] Roongaraya, P. & Boonyasuppayakorn, S. Chikungunya vaccines: an update in 2023. *Asian Pac. J. Allergy Immunol.* **41**, 1–11 (2023). 37029782 10.12932/AP-271222-1520

[35] Scholte, F. E. et al. Characterization of synthetic Chikungunya viruses based on the consensus sequence of recent E1-226V isolates. *PLoS ONE* **8**, e71047 (2013). 23936484 10.1371/journal.pone.0071047 PMC3731263

[36] Abramson, J. et al. Accurate structure prediction of biomolecular interactions with AlphaFold 3. *Nature* **630**, 493–500 (2024). 38718835 10.1038/s41586-024-07487-w PMC11168924

[37] Takatsu, K., Kouro, T. & Nagai, Y. Interleukin 5 in the link between the innate and acquired immune response. *Adv. Immunol.* **101**, 191–236 (2009). 19231596 10.1016/S0065-2776(08)01006-7

[38] Vazquez, M. I., Catalan-Dibene, J. & Zlotnik, A. B cells responses and cytokine production are regulated by their immune microenvironment. *Cytokine* **74**, 318–326 (2015). 25742773 10.1016/j.cyto.2015.02.007 PMC4475485

[39] Kam, Y.-W. et al. Early appearance of neutralizing immunoglobulin G3 antibodies is associated with Chikungunya virus clearance and long-term clinical protection. *J. Infect. Dis.* **205**, 1147–1154 (2012). 22389226 10.1093/infdis/jis033 PMC3295607

[40] Gardner, J. et al. Chikungunya virus arthritis in adult wild-type mice. *J. Virol.* **84**, 8021–8032 (2010). 20519386 10.1128/JVI.02603-09 PMC2916516

[41] Rawle, D. J., Hugo, L. E., Cox, A. L., Devine, G. J. & Suhrbier, A. Generating prophylactic immunity against arboviruses in vertebrates and invertebrates. *Nat. Rev. Immunol.* **24**, 621–636 (2024). 38570719 10.1038/s41577-024-01016-6

[42] Hallengärd, D. et al. Prime-boost immunization strategies against Chikungunya virus. *J. Virol.* **88**, 13333–13343 (2014). 25210177 10.1128/JVI.01926-14 PMC4249109

[43] López-Camacho, C. et al. Assessment of immunogenicity and neutralisation efficacy of viral-vectored vaccines against chikungunya virus. *Viruses* **11**, 322 (2019). 30987160 10.3390/v11040322 PMC6521086

[44] Folegatti, P. M. et al. A single dose of ChAdOx1 Chik vaccine induces neutralizing antibodies against four chikungunya virus lineages in a phase 1 clinical trial. *Nat. Commun.* **12**, 4636 (2021). 34330906 10.1038/s41467-021-24906-y PMC8324904

[45] Hallengärd, D. et al. Novel attenuated Chikungunya vaccine candidates elicit protective immunity in C57BL/6 mice. *J. Virol* **88**, 2858–2866 (2014). 24371047 10.1128/JVI.03453-13 PMC3958085

[46] Roy, C. J. et al. Chikungunya vaccine candidate is highly attenuated and protects nonhuman primates against telemetrically monitored disease following a single dose. *J. Infect. Dis.* **209**, 1891–1899 (2014). 24403555 10.1093/infdis/jiu014 PMC4038141

[47] August, A. et al. A phase 1 trial of lipid-encapsulated mRNA encoding a monoclonal antibody with neutralizing activity against Chikungunya virus. *Nat. Med.* **27**, 2224–2233 (2021). 34887572 10.1038/s41591-021-01573-6 PMC8674127

[48] Partidos, C. D. et al. Probing the attenuation and protective efficacy of a candidate chikungunya virus vaccine in mice with compromised interferon (IFN) signaling. *Vaccine* **29**, 3067–3073 (2011). 21300099 10.1016/j.vaccine.2011.01.076 PMC3081687

[49] Szurgot, I., Ljungberg, K., Kümmerer, B. M. & Liljeström, P. Infectious RNA vaccine protects mice against chikungunya virus infection. *Sci. Rep.* **10**, 21076 (2020). 33273501 10.1038/s41598-020-78009-7 PMC7712826

[50] Couderc, T. et al. Prophylaxis and therapy for Chikungunya virus infection. *J. Infect. Dis.* **200**, 516–523 (2009). 19572805 10.1086/600381 PMC7109959

[51] Fox, J. M. et al. Optimal therapeutic activity of monoclonal antibodies against chikungunya virus requires Fc-FcγR interaction on monocytes. *Sci. Immunol.* **4**, eaav5062 (2019). 30796092 10.1126/sciimmunol.aav5062 PMC6698136

[52] Yoon, I.-K. et al. High rate of subclinical chikungunya virus infection and association of neutralizing antibody with protection in a prospective cohort in the Philippines. *PLoS Negl. Trop. Dis.* **9**, e0003764 (2015). 25951202 10.1371/journal.pntd.0003764 PMC4423927

[53] Cherian, N. et al. Strategic considerations on developing a CHIKV vaccine and ensuring equitable access for countries in need. *npj Vaccines* **8**, 123 (2023). 37596253 10.1038/s41541-023-00722-x PMC10439111

[54] Bao, H. et al. Nonstructural protein 2 (nsP2) of Chikungunya virus (CHIKV) enhances protective immunity mediated by a CHIKV envelope protein expressing DNA vaccine. *Viral Immunol.* **26**, 75–83 (2013). 23409931 10.1089/vim.2012.0061 PMC4845693

[55] Pratheek, B., Suryawanshi, A. R., Chattopadhyay, S. & Chattopadhyay, S. In silico analysis of MHC-I restricted epitopes of Chikungunya virus proteins: Implication in understanding anti-CHIKV CD8+ T cell response and advancement of epitope based immunotherapy for CHIKV infection. *Infect. Genet. Evol.* **31**, 118–126 (2015). 25643869 10.1016/j.meegid.2015.01.017

[56] Roques, P. et al. Attenuated and vectored vaccines protect nonhuman primates against Chikungunya virus. *JCI Insight* **2**, e83527 (2017). 28352649 10.1172/jci.insight.83527 PMC5358498

