# Supplementary material for: CHIKV mRNA vaccines encoding conserved structural/envelope proteins confer broad cross-lineage protection against infection
Source: Signal Transduct Target Ther. 2025 Mar 28;10:98. doi: 10.1038/s41392-025-02182-2 (PMC11950367; doi:10.1038/s41392-025-02182-2)
Supplement: Supplementary file 1 — Revised 4 supplementary information [file 41392_2025_2182_MOESM1_ESM.docx]

**Supplementary Materials**

CHIKV mRNA vaccines encoding conserved structural/envelope proteins confer broad cross-lineage protection against infection.

Xiaoming Liang, Yanan Zhou, Yun Yang, Qianqian Li, Junbin Wang, Bai Li, Hao Yang, Cong Tang, Wenhai Yu, Haixuan Wang, Qing Huang, Hongyu Chen, Yuhuan Yan, Ran An, Dongdong Lin, Wenqi Quan, Yong Zhang, Yanwen Li, Xuena Du, Yuxia Yuan, Longhai Yuan, Jian Zhou, Qiangming Sun, Youchun Wang, Shuaiyao Lu

**This PDF file includes:**

Materials and Methods

Figure 1-11

DNA sequences of the vaccines

**Materials and Methods**

**Sequence alignment and plasmid construction**

After downloading the viral sequences from the NCBI Virus database, sequence alignment was performed using MAGE 11software, followed by phylogenetic tree construction using MAGE11, and the evolutionary tree was beautified using iTOL. Antigen sequences were codon-optimized and synthesized. The T7 promoter, 5’UTR, 3’UTR, and PolyA were constructed into the PUC57 plasmid, then the antigen sequence was constructed onto the vector through enzymatic digestion sites NcoI and XhoI or by homologous recombination method.

**Structural Prediction**

Referring to the 'The complex structure of CHIKV envelope glycoprotein bound to human MXRA8' (PDB: 6JO8) sequence in the PDB database, AlphaFold3 is utilized to predict the protein structure of the CHIKV vaccine and reference virus in complex with the human MXRA8 trimer. The resulting CIF crystal file is then subjected to visualization analysis using PyMOL. Protein-protein interaction analysis is conducted using the internal PyMOL script 'interfaceResidues' with a cutoff value of 0.75, yielding molecular docking images and interaction sites.

**Cell immunofluorescence**

Vero cells were cultured in a 24-well plate until they reached 70% confluence. The medium was then replaced with Opti-MEM, and 1 μg of each packaged mRNA was added to different wells. The mCV-1, mCV-2, and mock control groups were each performed in triplicate. After 6 hours, the medium was replaced with complete growth medium (DMEM). After 24 hours, the cells were washed three times with PBS, fixed with 4% paraformaldehyde, and permeabilized with 1% Triton X-100 following PBS washing. Blocking was performed using goat serum, and the cells were incubated with E1 antibody overnight at 4°C. Subsequently, the cells were washed with 0.5% PBST (0.5% Tween-20 in PBS), incubated with goat anti-rabbit secondary antibody (Abcam, #AB150081) at room temperature for 1 hour, and mounted with a medium containing DAPI. Finally, imaging was performed using fluorescence microscopy (Thermo Scientific, DMi8)

**T cell flow cytometry analysis**

As previously described, PBMCs were thawed, counted, and 2x10^7^ cells per sample were stimulated with E2 protein (final concentration 2 μg/ml) in R10 medium (1640 medium + 10% FBS + 1% Pen-Strep antibiotic), and cultured at 37°C for 3 hours. Golgi Stop transport inhibitor cocktail (BD, #554724) was added according to the manufacturer's instructions and incubated for an additional 6 hours. After washing with PBS, cells were stained for viability(BD, #565388) according to the manufacturer's instructions, washed with PBS, and resuspended using Fc block (BD, Cat:553141). Subsequently, cells were stained with antibodies CD3(Biolegend, #100227), CD4 (Biolegend, #100433), and CD8 (Biolegend, #100761) in staining buffer (PBS supplemented with 2.5% FBS) for 20 minutes, followed by fixation and permeabilization using BD Cytoperm Fixation/Permeabilization solution Kit(BD, 554723) according to the manufacturer's instructions. Cells were washed in Perm/Wash buffer, then stained intracellularly with the following antibodies (30 minutes, RT): IL-2 (Biolegend, #503805), IL-4 (Biolegend, #504103), IFN-γ (Biolegend, #505810), TNF-α (Biolegend, #506338). After washing with perm/wash, cells were resuspended in staining buffer and acquired on a BD Celesta flow cytometer.

**Mouse vaccination and samples collection**

For BALB/c mice, 6- to 8-week-old female BALB/c mice were utilized. Two vaccine candidates, mCV-1-LNP and mCV-2, were administered at doses of 4 μg, 8 μg, and 15 μg. An empty LNP was used as a placebo control group and administered via intramuscular injection using a 1 ml sterile syringe. Two weeks later, a booster dose was given to enhance the immune response. Changes in body temperature and weight were monitored after immunization. Serum samples were collected from immunized mice on days 7, 14, 21, and 28 to measure E2-specific immunoglobulin GMT and neutralizing antibodies. Splenocytes were collected on day 28 for Elispots and flow cytometry experiments.

For A129 mice, 6- to 8-week-old A129 mice (equal numbers of males and females) were used. Two vaccine candidates, mCV-1-LNP and mCV-2-LNP, were administered at doses of 4 μg and 15 μg. An empty LNP was used as a placebo control group and administered via intramuscular injection using a 1 ml sterile syringe. Two weeks later, a booster dose was given to enhance the immune response. Serum samples were collected from immunized mice on days 7, 14, 21, and 28 to measure E2-specific immunoglobulin GMT and neutralizing antibodies.

**Mouse challenge experiments**

For BALB/c mice, body temperature, body weight, and joint widtch and bredth were measured 42 days after primary immunization. A total of 50 μL of virus solution (10^7^ pfu/mL, total dose of 10^6^ per mouse) was injected subcutaneously at the left and right joints ankle joints of each mouse using a 50 μL microsyringe. Subsequently, on days 1, 3, 5, and 7 post-infection, blood samples were collected via tail vein for virus viremia detection, along with measurements of body temperature, body weight, and joint widtch and bredth. On the seventh day, all mice were euthanized, and tissues including heart, liver, spleen, lung, kidney, brain, left hind limb muscle, left forelimb joint, left hind limb joint, duodenum, rectum, and uterus were collected for immunofluorescence assays, histopathological section preparation, and RT-qPCR.

For A129 mice, body temperature, body weight, and joint widtch and bredth were measured 42 days after primary immunization. A total of 50 μL of virus solution (104 pfu/mL, total dose of 10^3^ per mouse) was injected into the left and right ankle joints of each mouse using a 50 μL microsyringe. Subsequently, body temperature, body weight, joint widtch and bredth were measured four days post-infection, followed by blood collection via tail vein. Blood collection was repeated on day 14, and euthanasia was performed on day 21, with tissue collection (similar to BALB/c mice) for virus load detection.

**Tissues immunofluorescence**

Muscle and liver tissues collected 24 hours post-immunization (or tissues on 7 days post-viral infection) were fixed in 4% paraformaldehyde solution for 72 hours, embedded in paraffin, and sectioned at 5 μm thickness. Deparaffinization was performed by three washes in xylene followed by rehydration in ethanol series (100%, 95%, 85%, 75%) and distilled water/PBS washes. Sections were subjected to antigen retrieval by heating in antigen retrieval solution at 100°C, followed by cooling for 20 minutes after three cycles of heating for 3 minutes each. After washing thrice with PBS, sections were permeabilized with 0.5% TritonX-100 PBS solution at room temperature for 30 minutes, blocked with goat serum for 1 hour, and then incubated overnight at 4°C with primary antibody against E1 protein. Following three washes with PBST, sections were incubated in the dark with fluorescence secondary antibodies for 1 hour. After PBST washing, sections were mounted with mounting medium containing DAPI and scanned using 3DHISTECH for analysis.

**Inactivation of CHIKV virus.**

CHIKVs were passaged in Vero cells, and the virus stock was aliquoted and titrated to plaque-forming units per milliliter (PFU/ml) in Vero cells using a plaque assay. The virus was placed at 56 for 30 minutes, followed by ultrafiltration using a 100 kDa ultrafilter. After washing with PBS, it was subjected to a second round of ultrafiltration, concentrating the solution to 1 ml before measuring the protein concentration.

**mRNA sequencing**

Whole blood samples were collected from mice 28 days after the initial immunization. Total RNAs were extracted from the blood using Trizol Reagent (cat. NO 15596026, Invitrogen). 2μg total RNAs were used for stranded RNA sequencing library preparation using KC-DigitalTM Stranded mRNA Library Prep Kit 464 for Illumina® (Catalog NO. DR08502, Wuhan Seqhealth Co., Ltd. China) following the manufacturer’s instruction. The library products corresponding to 200-500 bps were enriched, quantified and finally sequenced on DNBSEQ-T7 sequencer (MGI Tech Co., 467 Ltd. China) with PE150 model mRNA sequencing Statistical analysis

**BCR and TCR sequencing**

Whole blood samples were collected from mice 28 days after the initial immunization for BCR and TCR sequencing. RNA extraction testing was performed in the same way as for mRNA sequencing. About 2 μg total RNA of each sample were used for BCR and TCR sequencing library preparation using KC-DigitalTM Stranded BCR-seq Library Prep Kit for Illumina® 150 (Seqhealth Technology Co., Ltd., Wuhan, China, Cat. No. DT0815-02) following the manufacturer’s instruction. The library products corresponding to 250-500 bp were enriched, quantified and finally sequenced on NovaSeq (Illumina®). The sequence was mapped to the international ImMunoGeneTics (IMGT) database. And using MiXCR software (version 3.0.3) to obtain V , D, and J fragments, rearrangement, and CDR3 sequences.

**Rhesus Macaque Immunization Experiment:**

Nine male rhesus macaques, aged 4-8 years, were divided into three groups, with three animals per group. On Day 0 and Day 21, each group received an intramuscular immunization of 300 μg of either mCV-1, mCV-2, or an equivalent volume (total volume of 1 mL) of physiological saline at the quadriceps of the rhesus macaques. Serum samples were collected on Days 7, 14, 21, 28, and 35 post-initial immunization. On Day 35, blood was drawn to isolate PBMCs for Elispot assays and pseudovirus neutralization tests. A challenge experiment was conducted on Day 42.

**Rhesus Macaque Challenge Experiment:**

The challenge was performed on Day 42 post-initial immunization using a strain adapted to the species. A total of 2ⅹ10^7^ PFU of CHIKV virus was injected into the outer side of the left and right hind limbs of each monkey. Body temperature and weight changes were recorded on Days 0, 1, 3, 5, and 7, and whole blood was collected for viremia testing. On the seventh day, the nine rhesus macaques were euthanized, and the following tissues were collected for viral RNA load detection: tracheobronchial, heart, liver, spleen, lungs, hilar lymph nodes, kidneys, pancreas, brain, inguinal lymph nodes, submandibular lymph nodes, axillary lymph nodes, leg muscles, stomach, duodenum, jejunum, ileum, Peyer's patches, cecum, ascending colon, transverse colon, descending colon, rectum, and mesenteric lymph nodes. Hematoxylin and eosin staining was conducted to observe pathological damage.

**Isolation of PBMCs from Rhesus Macaques and Elispot Assays**

Whole blood from rhesus macaques was collected in anticoagulant centrifuge tubes (KWS, 210113). After centrifugation, the blood cells were diluted with PBS, and Ficoll-Paque density gradient medium was added for further centrifugation. The lymphocyte layer was extracted, washed, and counted. Elispot assays were conducted according to the manufacturer's protocol (Mabtech 3421M-4APW-2; 3445M-4APW-10; 3410-4APW-2), using E2 as the stimulant, with the remainder of the procedure following the mouse experiment protocol.

**Figure S1-S9**


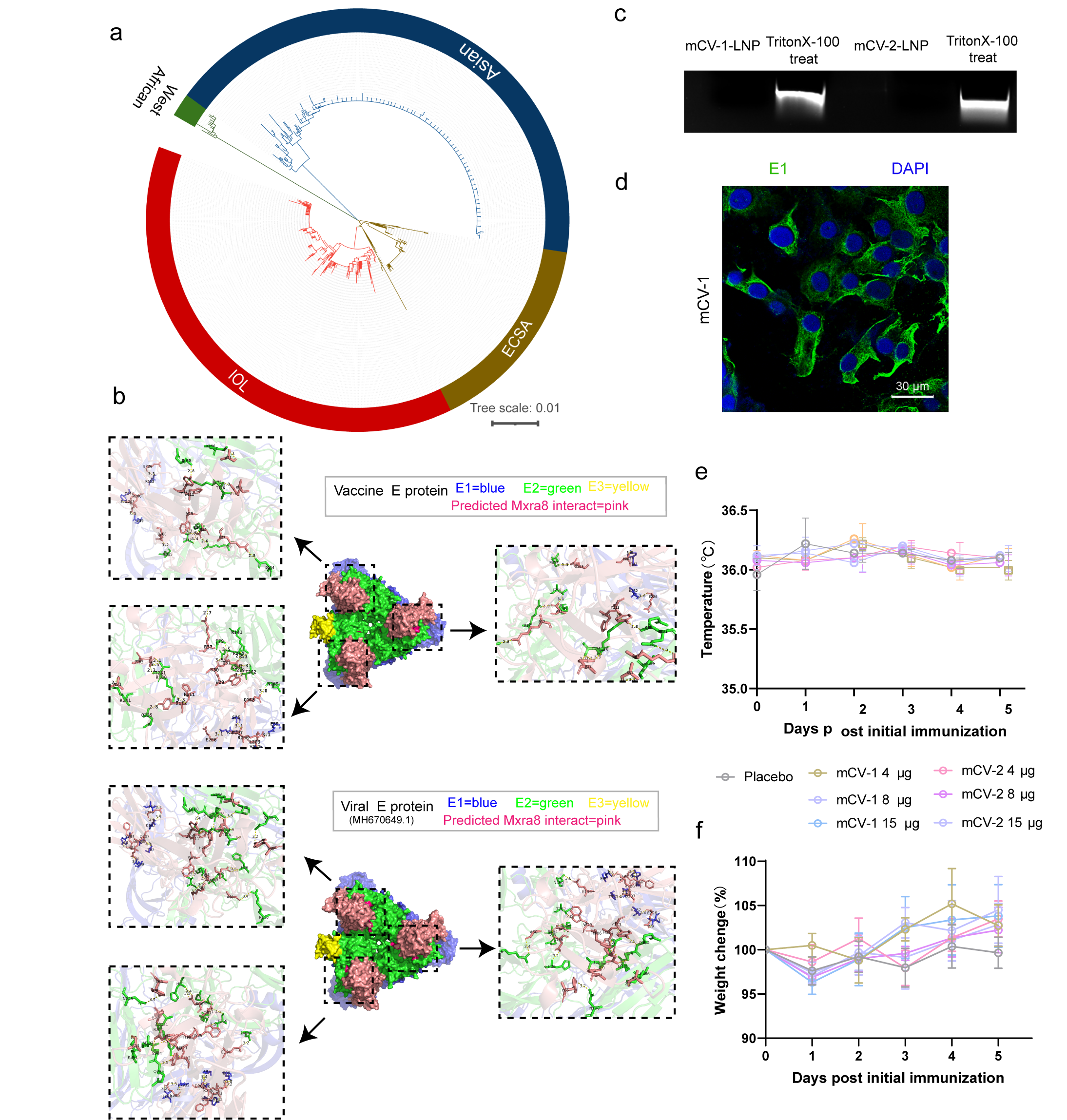


**Fig. 1.** a**,** The phylogenetic tree and lineage distribution of the virus strains used; b, Prediction of the interaction between E protein of vaccine and Mxra8 or E protein of virus and Mxra8; c, Verification of successful encapsulation through agarose gel electrophoresis and the observation of mRNA-LNP transfection-induced protein expression, the mCV-1-LNP and mCV-2-LNP lanes represent electrophoresis of mRNA encapsulated within LNPs, while the TritonX-100 treated lanes correspond to electrophoresis of mRNA released from the two encapsulated vaccines following Triton X-100 treatment ; d, mCV-LNP transfection of Vero cells immunofluorescence image, green represents E1protien, bule represents cell nucleus; e-f, Changes in body temperature (e) and weight (f) within 5 days post-immunization;


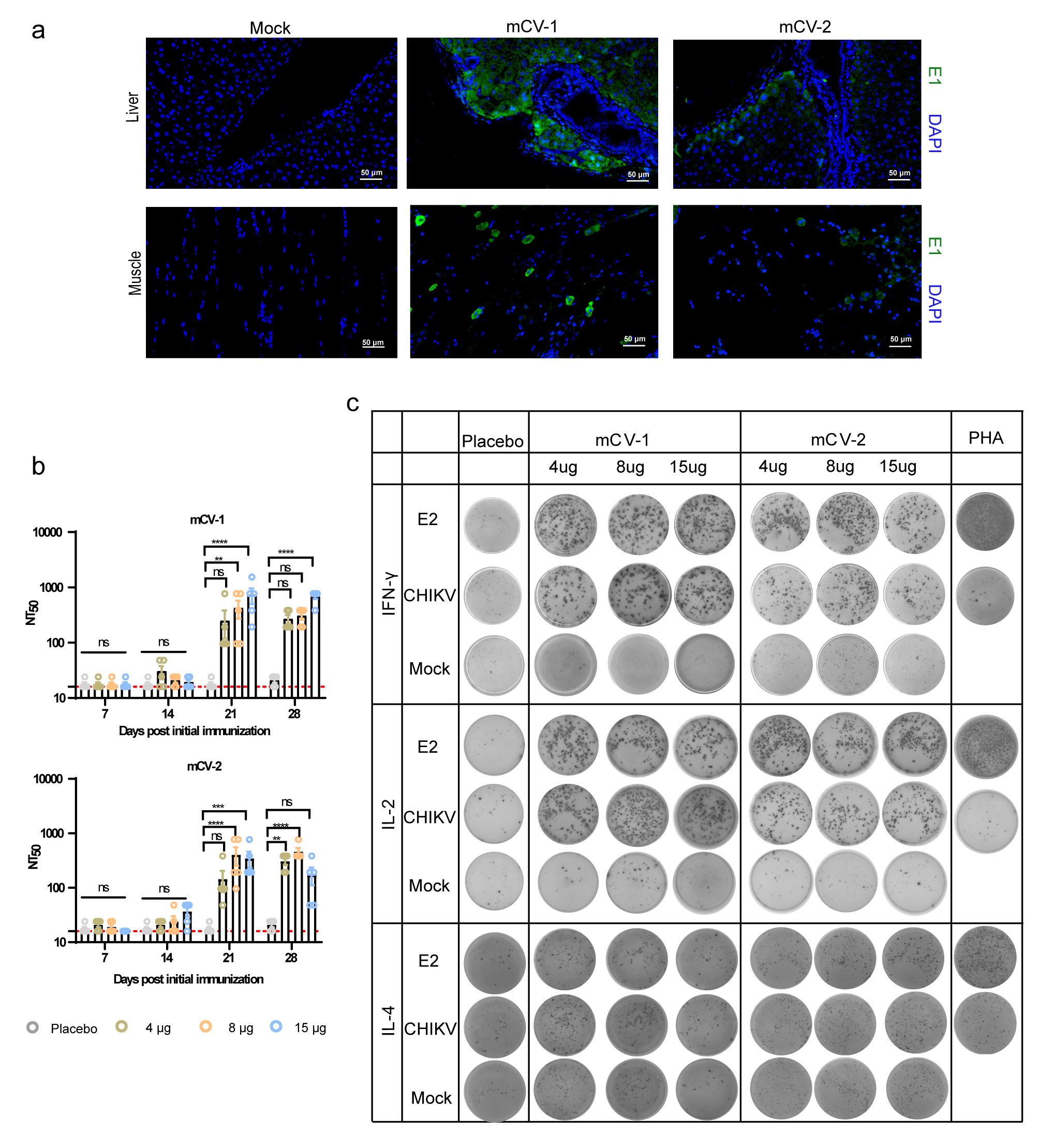


**Fig. 2**. a, Muscle and liver E1 protein expression at 24 hours post-immunization; b,The titers of neutralizing antibodies against the prototype strain (MH670649.1); c, The representative images of Elispot experiments (c).


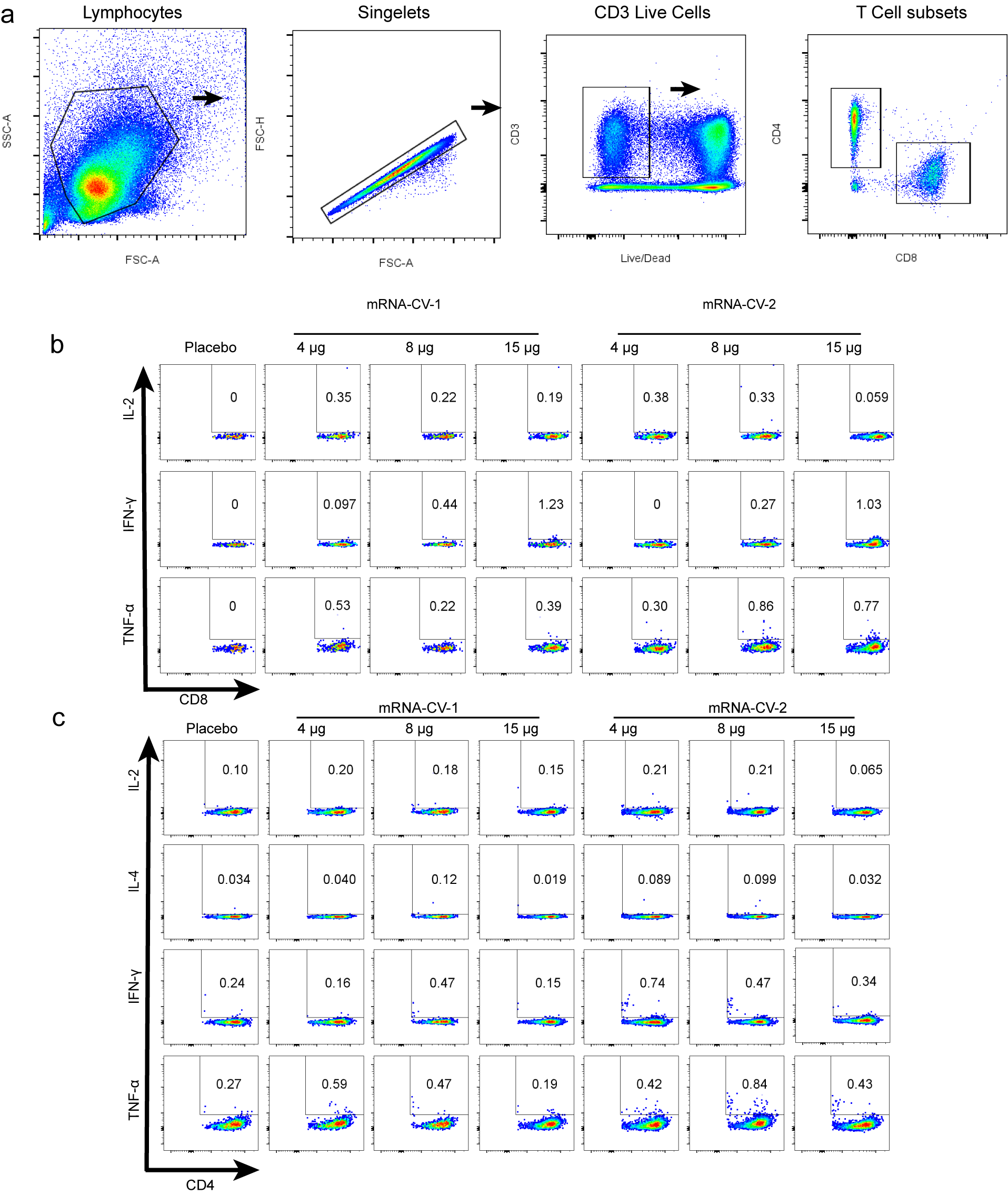


**Fig. 3**. Gating strategy for flow cytometry experiments(a) and Representative images for flow cytometry experiments (b).


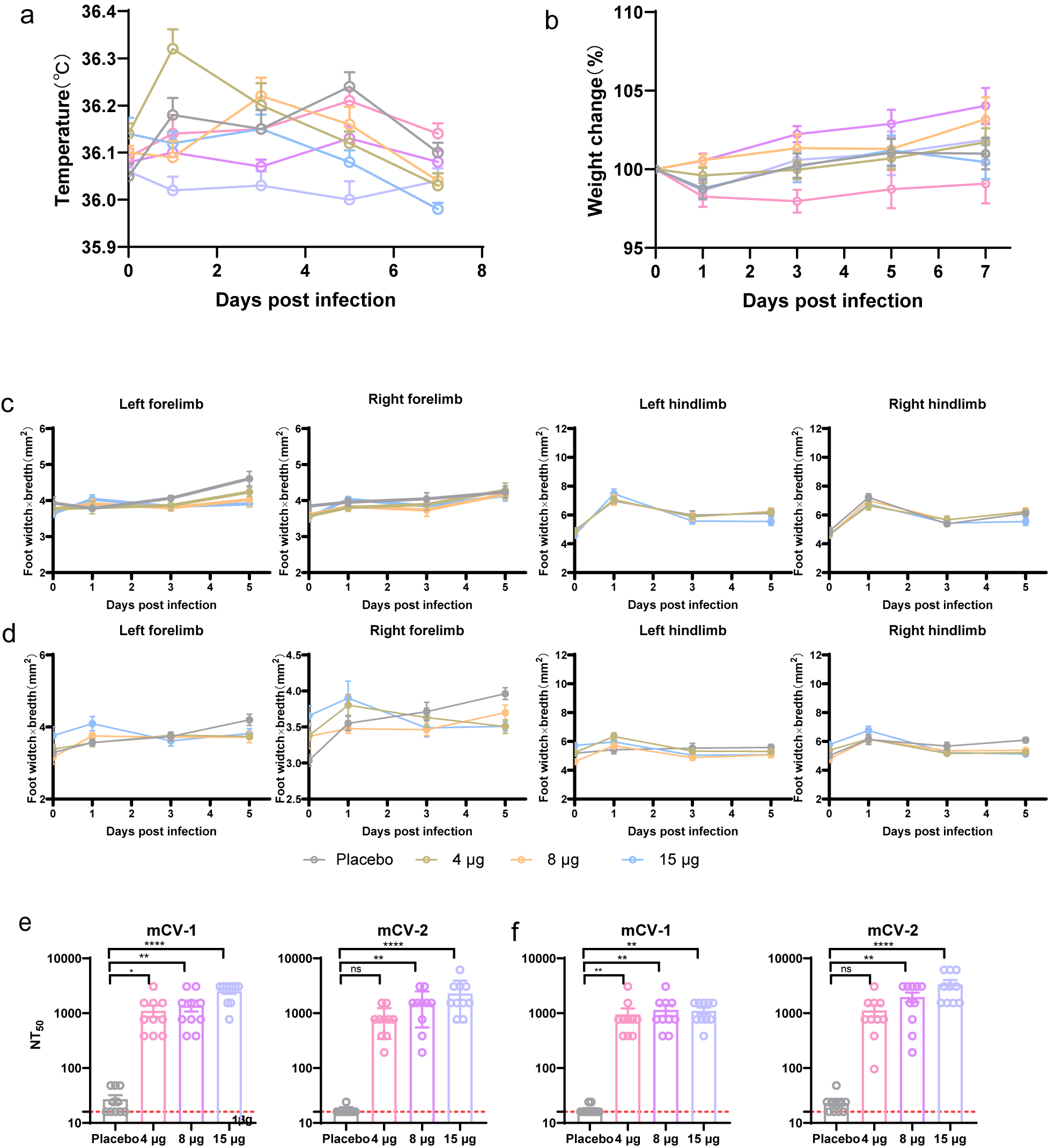


**Fig. 4.** Changes in body temperature (a) and weight (b) post-infection. C-d, Changes in joint swelling for mCV-1 (c) and mCV-2 (d); e-f, Neutralizing antibody levels after infection with the original strain (e) or the adapted strain (f). Statistical analysis was conducted using one-way ANOVA and Tukey’s multiple comparison test for bar graphs; *p < 0.05; **p < 0.01; ***p < 0.001; ****p < 0.0001; ns, not significant.


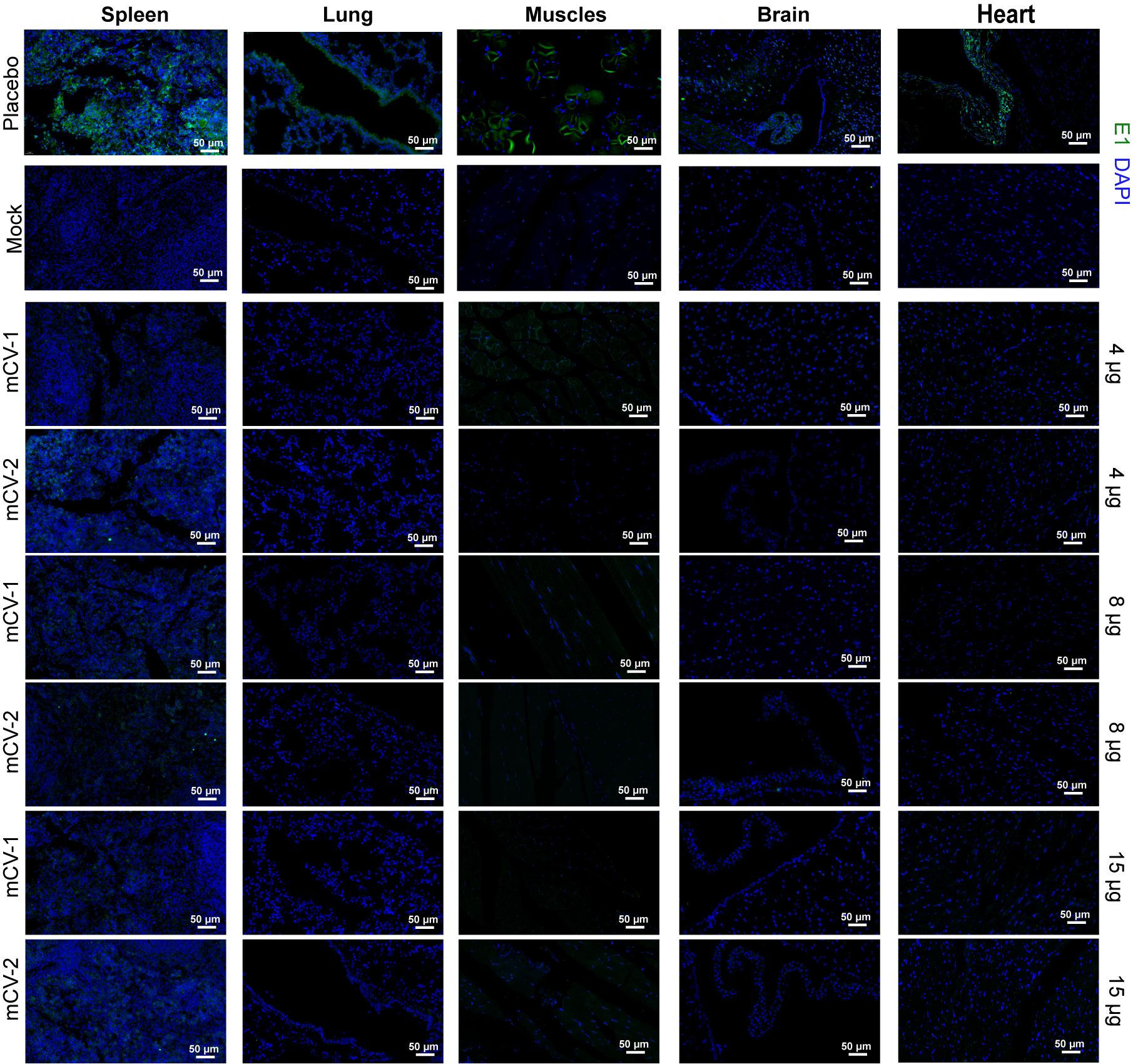


**Fig. 5**. Immunofluorescence of Various Tissues on the Seventh Day Post-Challenge in spleen, lung, muscles, brain and heart. The green indicates the E1 protien and blue indicates DAPI.


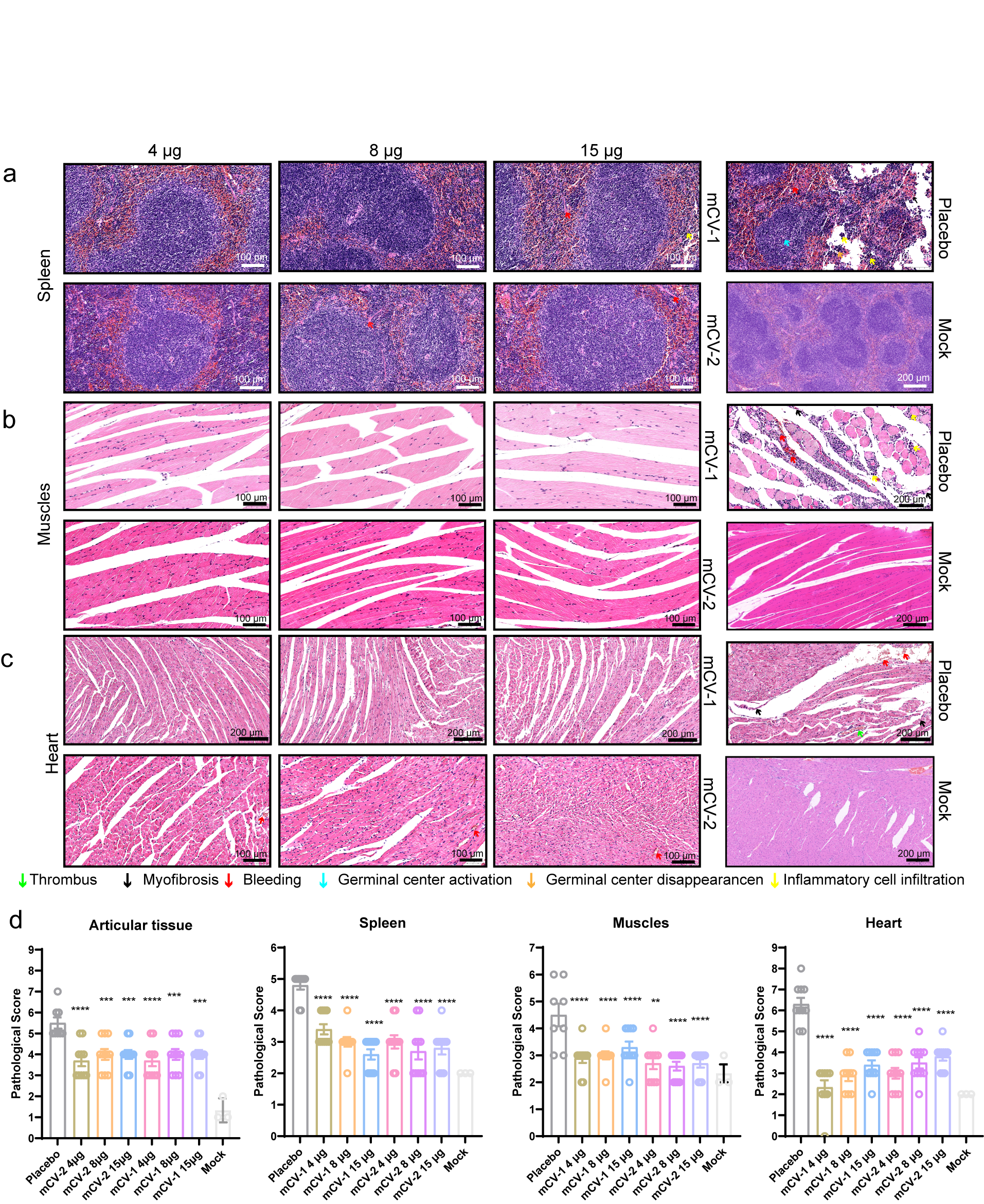


**Fig. 6**. Pathological tissue sections for the spleen (a), muscles (b) and heart (c); d, Pathological scores on the Seventh Day Post-Challenge for the joint tissue, spleen, muscles and heart; Statistical analysis was conducted using one-way ANOVA and Tukey’s multiple comparison test for bar graphs; *p < 0.05; **p < 0.01; ***p < 0.001; ****p < 0.0001; ns, not significant.


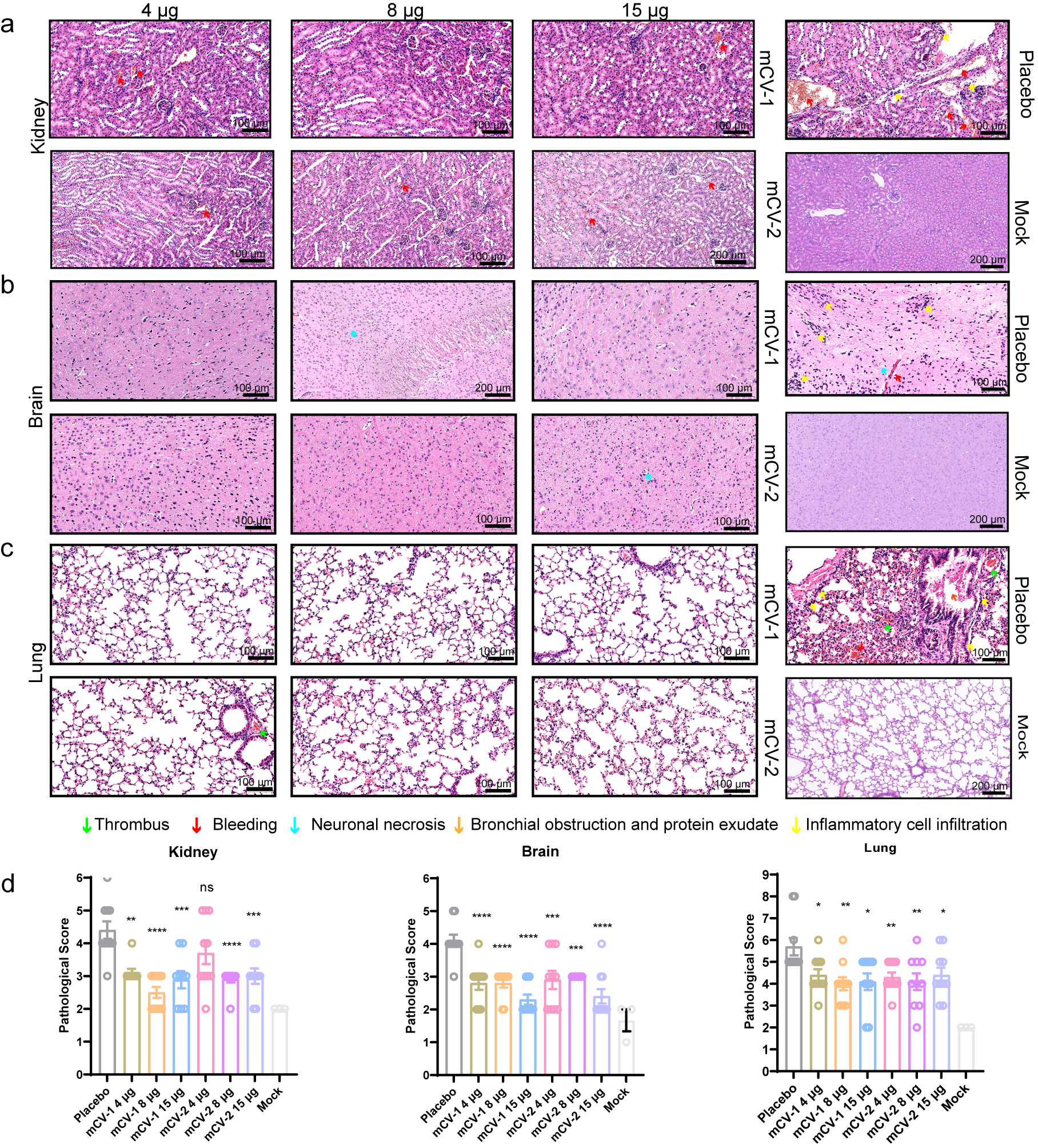


**Fig. 7**. Pathological tissue sections for the, kidney (a), brain (b) and Lung (c); d, Pathological scores on the Seventh Day Post-Challenge for the kidney, brain, and Lung; Statistical analysis was conducted using one-way ANOVA and Tukey’s multiple comparison test for bar graphs; *p < 0.05; **p < 0.01; ***p < 0.001; ****p < 0.0001; ns, not significant.


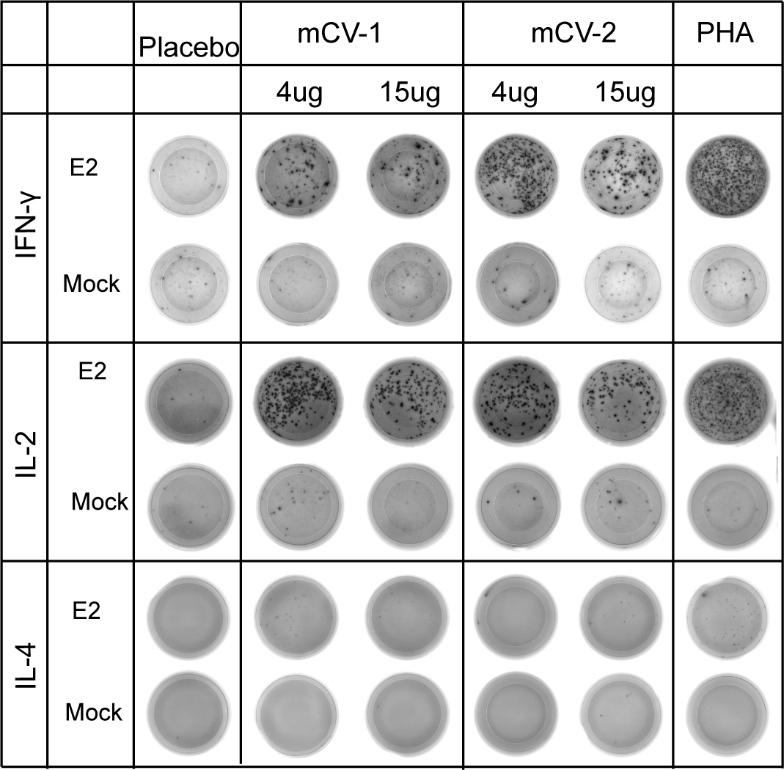


**Fig. 8** Representative images of Elispot experiments in A129 mice


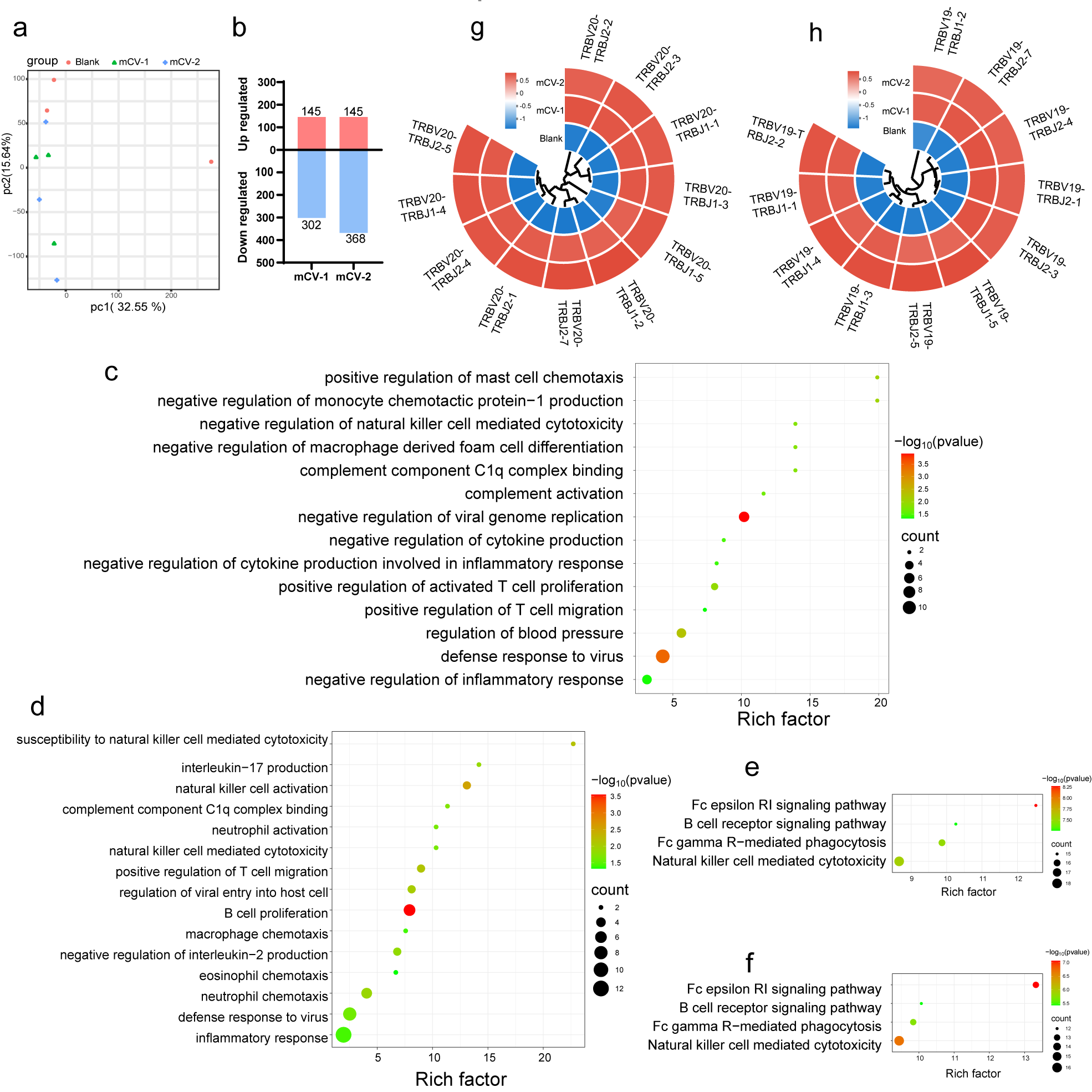


**Fig. 9**. a, PCA of transcriptome sequencing samples. b, Differential gene expression; c-d, GO enrichment analysis for mCV-1 and mCV-2, e-f, KEGG enrichment analysis for mCV-1 and mCV-2; g-h, Comparison of Gene Usage Frequencies of V19 and V20 Between the Vaccine Group and the Blank Group

**Fig. 10.** The E2-specific IgG3 binding antibody titers in BALB/c mice 28 days after the initial immunization (n=5). Statistical analysis was conducted using one-way ANOVA and Tukey’s multiple comparison test for bar graphs; *p < 0.05; **p < 0.01; ***p < 0.001; ****p < 0.0001; ns, not significant.


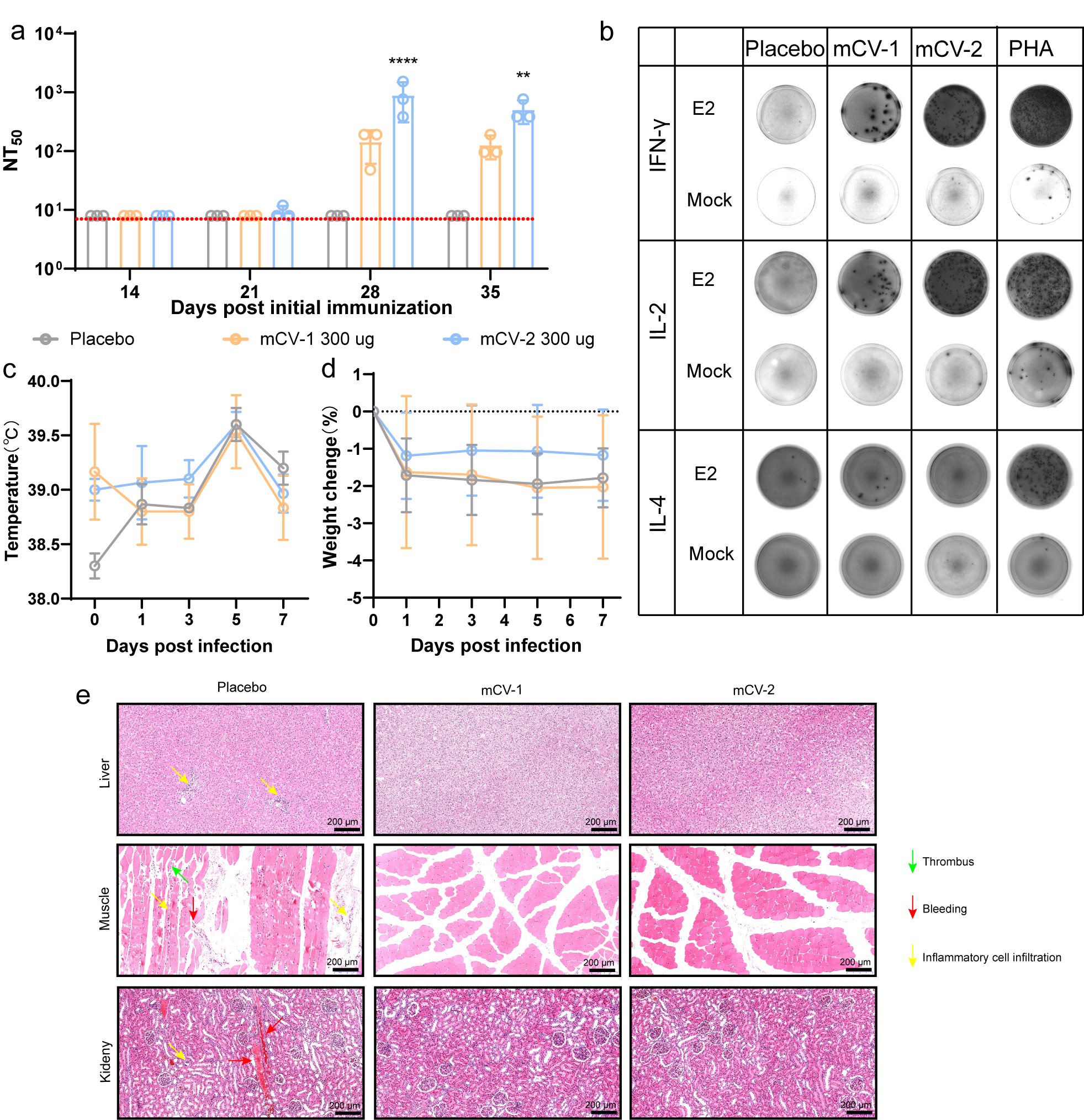


**Fig. 11.** The mCV-1 and mCV-2 vaccines protect rhesus macaques from challenge with CHIKV. a, The titers of neutralizing antibodies against the prototype strain (MH670649.1);b, Representative images of Elispot experiments in rhesus macaques; c, Changes in body temperature (c) and weight (d) post-infection; e, Pathological tissue sections for the liver, muscle and kidney.

**The DNA sequences of the vaccines：**

**mCV-1**：

**AGAATAAACTAGTATTCTTCTGGTCCCCACAGACTCAGAGAGAACCCGCCACCATGGAGTTCATCCCCACCCAGACCTTCTACAACAGAAGATACCAGCCCAGACCCTGGACCCCTAGACCTACAATCCAGGTGATTAGACCCAGACCCCGCCCTCAGAGACAGGCTGGACAGCTGGCTCAGCTGATTAGCGCTGTGAACAAACTGACCATGAGAGCCGTGCCCCAGCAGAAACCAAGAAGAAACAGAAAGAACAAGAAGCAGAAACAGAAACAGCAGGCCCCTCAGAACAACACAAACCAGAAGAAGCAGCCACCAAAGAAGAAGCCCGCCCAGAAGAAGAAGAAGCCAGGCAGAAGAGAAAGAATGTGCATGAAGATTGAGAACGACTGCATCTTCGAAGTGAAACACGAGGGCAAGGTGACCGGCTACGCTTGTCTGGTGGGAGATAAGGTGATGAAGCCAGCCCATGTGAAGGGAACCATCGACAACGCCGACCTGGCTAAACTGGCCTTCAAGAGAAGCAGCAAGTATGACCTGGAGTGCGCCCAGATTCCTGTGCACATGAAGTCTGACGCCAGCAAATTCACCCACGAGAAACCCGAAGGCTACTACAACTGGCACCACGGAGCCGTGCAGTACTCAGGAGGAAGATTCACCATCCCCACCGGCGCTGGAAAACCTGGAGATTCTGGAAGGCCTATTTTTGACAACAAGGGCAGAGTGGTGGCCATCGTGCTGGGAGGAGCTAATGAGGGAGCCAGAACAGCCCTGAGCGTGGTGACATGGAACAAGGACATCGTGACAAAGATTACACCCGAGGGCGCCGAGGAATGGTCACTGGCTATTCCAGTGATGTGCCTGCTGGCCAACACCACATTCCCCTGTTCCCAGCCTCCTTGCACCCCTTGTTGCTACGAAAAGGAACCCGAAGAAACACTGAGAATGCTGGAGGACAACGTGATGAGGCCTGGCTACTACCAGCTGCTGCAAGCTAGTCTGACCTGCTCCCCTCACAGACAGAGAAGGTCCACCAAAGACAACTTCAACGTGTACAAAGCCACCCGGCCATACCTGGCTCACTGTCCTGATTGCGGCGAAGGACACTCCTGTCACAGCCCTGTGGCTCTGGAAAGAATCAGAAACGAGGCCACCGACGGCACCCTGAAAATCCAGGTGAGCCTGCAAATCGGCATTAAAACCGACGACAGCCATGACTGGACCAAGCTGAGATACATGGATAACCACATGCCCGCCGACGCCGAAAGAGCTGGACTGTTCGTGAGGACCAGCGCTCCTTGTACCATCACCGGAACAATGGGTCACTTCATCCTGGCTAGATGCCCCAAGGGCGAAACCCTGACAGTGGGATTTACCGATAGCAGAAAAATCAGCCACAGCTGCACCCACCCCTTCCATCATGACCCACCTGTGATTGGACGCGAGAAGTTCCATAGCAGACCACAGCACGGAAAAGAACTGCCCTGCTCCACCTACGTGCAGTCAACAGCTGCCACCACCGAAGAAATCGAGGTGCACATGCCTCCTGATACCCCTGACAGAACCCTGATGTCACAGCAGAGCGGCAACGTGAAGATTACCGTGAACGGCCAGACCGTGCGCTACAAATGCAACTGCGGCGACAGCAACGAGGGCCTGACAACAACAGACAAAGTGATTAACAACTGCAAGGTGGACCAGTGTCACGCCGCTGTGACAAACCACAAGAAGTGGCAGTACAACAGCCCCCTGGTGCCTAGAAACGCCGAACTGGGAGACAGAAAAGGAAAGATTCACATCCCCTTCCCACTGGCCAACGTGACATGCAGAGTGCCAAAAGCCAGAAACCCCACCGTGACATACGGCAAAAACCAGGTGATTATGCTGCTGTACCCCGACCACCCCACCCTGCTGTCTTATAGAAACATGGGAGAGGAACCCAACTACCAGGAAGAATGGGTGACCCACAAGAAGGAAGTGGTGCTGACCGTGCCTACCGAGGGACTGGAAGTGACTTGGGGAAACAACGAACCCTACAAGTACTGGCCCCAGCTGTCCACAAACGGCACAGCTCATGGGCACCCACACGAAATCATCCTGTACTACTACGAACTGTACCCCACCATGACCGTGGTGGTGGTGTCAGTGGCTTCCTTCATCCTGCTGAGCATGGTGGGCGTGGCTGTGGGAATGTGTATGTGCGCTAGAAGGCGGTGCATCACCCCTTACGAACTGACTCCCGGCGCTACAGTGCCTTTCCTGCTGTCTCTGATTTGCTGCATCAGAACTGCCAAGGCTGCCACCTATCAGGAGGCTGCTGTGTACCTGTGGAACGAGCAGCAGCCTCTGTTCTGGCTGCAAGCTCTGATTCCCCTGGCCGCTCTGATTGTGCTGTGTAATTGCCTGAGACTGCTGCCCTGCTGCTGCAAAACCCTGGCTTTCCTGGCCGTGATGAGCGTGGGAGCCCATACAGTGTCCGCTTACGAGCACGTCACAGTGATTCCCAATACTGTGGGCGTGCCCTACAAAACCCTGGTGAACAGGCCCGGATATTCCCCTATGGTGCTGGAGATGGAACTGCTGTCCGTGACTCTGGAGCCCACACTGAGTCTGGACTACATCACCTGCGAATACAAGACCGTGATTCCCTCCCCTTACGTGAAATGTTGCGGCACCGCCGAGTGCAAAGACAAAAACCTGCCCGATTACAGCTGCAAAGTGTTCACCGGCGTGTACCCCTTCATGTGGGGAGGAGCTTATTGCTTCTGTGACGCCGAGAACACCCAGCTGAGCGAAGCTCATGTGGAGAAATCAGAAAGCTGTAAGACCGAATTTGCCAGCGCCTACAGAGCCCACACCGCTTCTGCTTCTGCCAAGCTGAGAGTGCTGTATCAGGGCAACAACATTACCGTGACCGCCTATGCTAACGGCGACCATGCTGTGACCGTGAAGGATGCTAAATTCATCGTGGGCCCCATGAGCAGTGCCTGGACACCTTTTGATAACAAGATTGTGGTGTACAAGGGCGATGTGTACAACATGGACTACCCTCCTTTCGGGGCCGGAAGGCCTGGTCAATTTGGAGATATTCAGTCCAGAACCCCTGAGAGCGAGGACGTGTACGCTAACACCCAGCTGGTGCTGCAAAGACCCGCTGCTGGAACTGTGCACGTCCCTTATAGTCAGGCCCCTAGCGGATTCAAGTACTGGCTGAAGGAAAGAGGAGCCAGCCTGCAACACACCGCTCCTTTTGGATGCCAGATTGCCACTAATCCCGTGCGGGCTATGAATTGCGCCGTGGGAAACATGCCAATTAGTATCGACATCCCCGACGCTGCCTTCACAAGAGTGGTGGATGCCCCTTCCCTGACAGACATGAGCTGTGAGGTGCCTGCTTGCACCCACTCATCTGATTTCGGCGGCGTGGCTATCATCAAGTATGCCGCTTCCAAGAAGGGGAAATGCGCTGTGCACAGTATGACCAATGCCGTGACCATCAGGGAAGCCGAAATTGAAGTGGAGGGCAACTCTCAGCTGCAAATCTCCTTCAGCACCGCCCTGGCTTCCGCTGAATTTAGAGTGCAGGTGTGCAGCACCCAGGTGCATTGTGCTGCTGAGTGTCATCCTCCTAAAGACCATATCGTGAACTACCCCGCCAGCCACACCACACTGGGAGTGCAGGATATTAGTGCCACAGCTATGTCTTGGGTGCAGAAAATTACCGGCGGAGTGGGACTGGTGGTGGCTGTGGCTGCTCTGATTCTGATTGTGGTGCTGTGCGTGTCCTTCAGCAGGCATTAACTCGAGCTGGTACTGCATGCACGCAATGCTAGCTGCCCCTTTCCCGTCCTGGGTACCCCGAGTCTCCCCCGACCTCGGGTCCCAGGTATGCTCCCACCTCCACCTGCCCCACTCACCACCTCTGCTAGTTCCAGACACCTCCCAAGCACGCAGCAATGCAGCTCAAAACGCTTAGCCTAGCCACACCCCCACGGGAAACAGCAGTGATTAACCTTTAGCAATAAACGAAAGTTTAACTAAGCTATACTAACCCCAGGGTTGGTCAATTTCGTGCCAGCCACACCCTGGAGCTAGCAAAAAAAAAAAAAAAAAAAAAAAAAAAAAAGCATATGACTAAAAAAAAAAAAAAAAAAAAAAAAAAAAAAAAAAAAAAAAAAAAAAAAAAAAAAAAAAAAAAAAAAAAAAA**

**mCV-2:**

**AGAATAAACTAGTATTCTTCTGGTCCCCACAGACTCAGAGAGAACCCGCCACCATGTCACTGGCTATTCCAGTGATGTGCCTGCTGGCCAACACCACATTCCCCTGTTCCCAGCCTCCTTGCACCCCTTGTTGCTACGAAAAGGAACCCGAAGAAACACTGAGAATGCTGGAGGACAACGTGATGAGGCCTGGCTACTACCAGCTGCTGCAAGCTAGTCTGACCTGCTCCCCTCACAGACAGAGAAGGTCCACCAAAGACAACTTCAACGTGTACAAAGCCACCCGGCCATACCTGGCTCACTGTCCTGATTGCGGCGAAGGACACTCCTGTCACAGCCCTGTGGCTCTGGAAAGAATCAGAAACGAGGCCACCGACGGCACCCTGAAAATCCAGGTGAGCCTGCAAATCGGCATTAAAACCGACGACAGCCATGACTGGACCAAGCTGAGATACATGGATAACCACATGCCCGCCGACGCCGAAAGAGCTGGACTGTTCGTGAGGACCAGCGCTCCTTGTACCATCACCGGAACAATGGGTCACTTCATCCTGGCTAGATGCCCCAAGGGCGAAACCCTGACAGTGGGATTTACCGATAGCAGAAAAATCAGCCACAGCTGCACCCACCCCTTCCATCATGACCCACCTGTGATTGGACGCGAGAAGTTCCATAGCAGACCACAGCACGGAAAAGAACTGCCCTGCTCCACCTACGTGCAGTCAACAGCTGCCACCACCGAAGAAATCGAGGTGCACATGCCTCCTGATACCCCTGACAGAACCCTGATGTCACAGCAGAGCGGCAACGTGAAGATTACCGTGAACGGCCAGACCGTGCGCTACAAATGCAACTGCGGCGACAGCAACGAGGGCCTGACAACAACAGACAAAGTGATTAACAACTGCAAGGTGGACCAGTGTCACGCCGCTGTGACAAACCACAAGAAGTGGCAGTACAACAGCCCCCTGGTGCCTAGAAACGCCGAACTGGGAGACAGAAAAGGAAAGATTCACATCCCCTTCCCACTGGCCAACGTGACATGCAGAGTGCCAAAAGCCAGAAACCCCACCGTGACATACGGCAAAAACCAGGTGATTATGCTGCTGTACCCCGACCACCCCACCCTGCTGTCTTATAGAAACATGGGAGAGGAACCCAACTACCAGGAAGAATGGGTGACCCACAAGAAGGAAGTGGTGCTGACCGTGCCTACCGAGGGACTGGAAGTGACTTGGGGAAACAACGAACCCTACAAGTACTGGCCCCAGCTGTCCACAAACGGCACAGCTCATGGGCACCCACACGAAATCATCCTGTACTACTACGAACTGTACCCCACCATGACCGTGGTGGTGGTGTCAGTGGCTTCCTTCATCCTGCTGAGCATGGTGGGCGTGGCTGTGGGAATGTGTATGTGCGCTAGAAGGCGGTGCATCACCCCTTACGAACTGACTCCCGGCGCTACAGTGCCTTTCCTGCTGTCTCTGATTTGCTGCATCAGAACTGCCAAGGCTGCCACCTATCAGGAGGCTGCTGTGTACCTGTGGAACGAGCAGCAGCCTCTGTTCTGGCTGCAAGCTCTGATTCCCCTGGCCGCTCTGATTGTGCTGTGTAATTGCCTGAGACTGCTGCCCTGCTGCTGCAAAACCCTGGCTTTCCTGGCCGTGATGAGCGTGGGAGCCCATACAGTGTCCGCTTACGAGCACGTCACAGTGATTCCCAATACTGTGGGCGTGCCCTACAAAACCCTGGTGAACAGGCCCGGATATTCCCCTATGGTGCTGGAGATGGAACTGCTGTCCGTGACTCTGGAGCCCACACTGAGTCTGGACTACATCACCTGCGAATACAAGACCGTGATTCCCTCCCCTTACGTGAAATGTTGCGGCACCGCCGAGTGCAAAGACAAAAACCTGCCCGATTACAGCTGCAAAGTGTTCACCGGCGTGTACCCCTTCATGTGGGGAGGAGCTTATTGCTTCTGTGACGCCGAGAACACCCAGCTGAGCGAAGCTCATGTGGAGAAATCAGAAAGCTGTAAGACCGAATTTGCCAGCGCCTACAGAGCCCACACCGCTTCTGCTTCTGCCAAGCTGAGAGTGCTGTATCAGGGCAACAACATTACCGTGACCGCCTATGCTAACGGCGACCATGCTGTGACCGTGAAGGATGCTAAATTCATCGTGGGCCCCATGAGCAGTGCCTGGACACCTTTTGATAACAAGATTGTGGTGTACAAGGGCGATGTGTACAACATGGACTACCCTCCTTTCGGGGCCGGAAGGCCTGGTCAATTTGGAGATATTCAGTCCAGAACCCCTGAGAGCGAGGACGTGTACGCTAACACCCAGCTGGTGCTGCAAAGACCCGCTGCTGGAACTGTGCACGTCCCTTATAGTCAGGCCCCTAGCGGATTCAAGTACTGGCTGAAGGAAAGAGGAGCCAGCCTGCAACACACCGCTCCTTTTGGATGCCAGATTGCCACTAATCCCGTGCGGGCTATGAATTGCGCCGTGGGAAACATGCCAATTAGTATCGACATCCCCGACGCTGCCTTCACAAGAGTGGTGGATGCCCCTTCCCTGACAGACATGAGCTGTGAGGTGCCTGCTTGCACCCACTCATCTGATTTCGGCGGCGTGGCTATCATCAAGTATGCCGCTTCCAAGAAGGGGAAATGCGCTGTGCACAGTATGACCAATGCCGTGACCATCAGGGAAGCCGAAATTGAAGTGGAGGGCAACTCTCAGCTGCAAATCTCCTTCAGCACCGCCCTGGCTTCCGCTGAATTTAGAGTGCAGGTGTGCAGCACCCAGGTGCATTGTGCTGCTGAGTGTCATCCTCCTAAAGACCATATCGTGAACTACCCCGCCAGCCACACCACACTGGGAGTGCAGGATATTAGTGCCACAGCTATGTCTTGGGTGCAGAAAATTACCGGCGGAGTGGGACTGGTGGTGGCTGTGGCTGCTCTGATTCTGATTGTGGTGCTGTGCGTGTCCTTCAGCAGGCATTAACTCGAGCTGGTACTGCATGCACGCAATGCTAGCTGCCCCTTTCCCGTCCTGGGTACCCCGAGTCTCCCCCGACCTCGGGTCCCAGGTATGCTCCCACCTCCACCTGCCCCACTCACCACCTCTGCTAGTTCCAGACACCTCCCAAGCACGCAGCAATGCAGCTCAAAACGCTTAGCCTAGCCACACCCCCACGGGAAACAGCAGTGATTAACCTTTAGCAATAAACGAAAGTTTAACTAAGCTATACTAACCCCAGGGTTGGTCAATTTCGTGCCAGCCACACCCTGGAGCTAGCAAAAAAAAAAAAAAAAAAAAAAAAAAAAAAGCATATGACTAAAAAAAAAAAAAAAAAAAAAAAAAAAAAAAAAAAAAAAAAAAAAAAAAAAAAAAAAAAAAAAAAAAAAAA**
